# Interpretable prediction of macrotrabecular-massive HCC and recurrence-free survival by integrating MRI LI-RADS features with deep learning habitat radiomics

**DOI:** 10.1186/s41747-026-00795-y

**Published:** 2026-09-08

**Authors:** Mengwei Zhang, Qian Wu, Mo Zhu, Tao Zhang, Chunyan Gu, Wenhao Gu, Mingzhan Du, Hailin Shen, Chunhong Hu, Yan Yang, Ximing Wang, Yixing Yu

**Affiliations:** 1 Department of Radiology, The First Affiliated Hospital of Soochow University, Suzhou, China; 2 Department of Radiology, Nantong Third People’s Hospital, Affiliated Nantong Hospital 3 of Nantong University, Nantong, China; 3 Department of Pathology, Nantong Third People’s Hospital, Affiliated Nantong Hospital 3 of Nantong University, Nantong, China; 4 Department of Radiology, The First People’s Hospital of Taicang, Suzhou, China; 5 Department of Pathology, The First Affiliated Hospital of Soochow University, Suzhou, China; 6 Department of Radiology, Suzhou Kowloon Hospital, Shanghai Jiaotong University School of Medicine, Suzhou, China

**Keywords:** Carcinoma (hepatocellular), Deep learning, Magnetic resonance imaging, Nomograms, Radiomics

## Abstract

**Objective:**

Macrotrabecular-massive (MTM+) hepatocellular carcinoma (HCC) is associated with poor prognosis and early recurrence. We developed and validated a nomogram integrating magnetic resonance imaging LI-RADS features with deep learning (DL) habitat radiomics for preoperative prediction of MTM + HCC and stratifying patients according to recurrence-free survival (RFS).

**Materials and methods:**

In this retrospective multicenter study, 607 patients with early-stage HCC who underwent curative-intent surgical resection (mean age ± standard deviation, 59.6 ± 10.6 years; 474 males, 133 females) were divided into the training (*n* = 304), internal validation (*n* = 131), and external (*n* = 172) test sets. Liver Imaging Reporting and Data System (LI-RADS) features, LI-RADS categorization, and clinical features were analyzed. Finally, a nomogram integrating LI-RADS features, habitat radiomics, and DL features was developed using multivariate logistic regression. Multivariable Cox regression analyses were performed to identify independent prognostic factors.

**Results:**

At multivariable analysis, habitat radiomics score, DL score, alpha-fetoprotein (AFP), alanine transferase (ALT), and fat mass were independent predictors of MTM + HCC. The DL habitat radiomics nomogram demonstrated powerful performance with areas under the receiver operating characteristic curve (AUCs) of 0.897 (95% confidence interval [CI]: 0.854–0.940), 0.757 (95% CI: 0.674–0.827), and 0.858 (95% CI: 0.797–0.906) in the training, internal validation, and external test sets, respectively. After multivariable analysis, AFP (*p* < 0.001) and nomogram-predicted MTM state (*p* = 0.039) resulted as independent prognostic factors.

**Conclusion:**

The nomogram integrating LI-RADS features, habitat radiomics, and DL features accurately identified patients with MTM + HCC and stratified patients by RFS.

**Key Points:**

- ***Question*** Preoperative integrative methods for accurate prediction of MTM + HCC and postoperative recurrence risk stratification remain insufficient.
- ***Findings*** The nomogram integrating LI-RADS features and DL habitat radiomics effectively diagnosed MTM + HCC and showed superior diagnostic performance compared to the Clinical-radiologic model.
- ***Relevance statement*** The nomogram provides a robust, non-invasive tool for preoperative identification of MTM + HCC and recurrence risk stratification, facilitating surgical planning and postoperative surveillance strategies.

**Graphical Abstract:**

**Figure:**
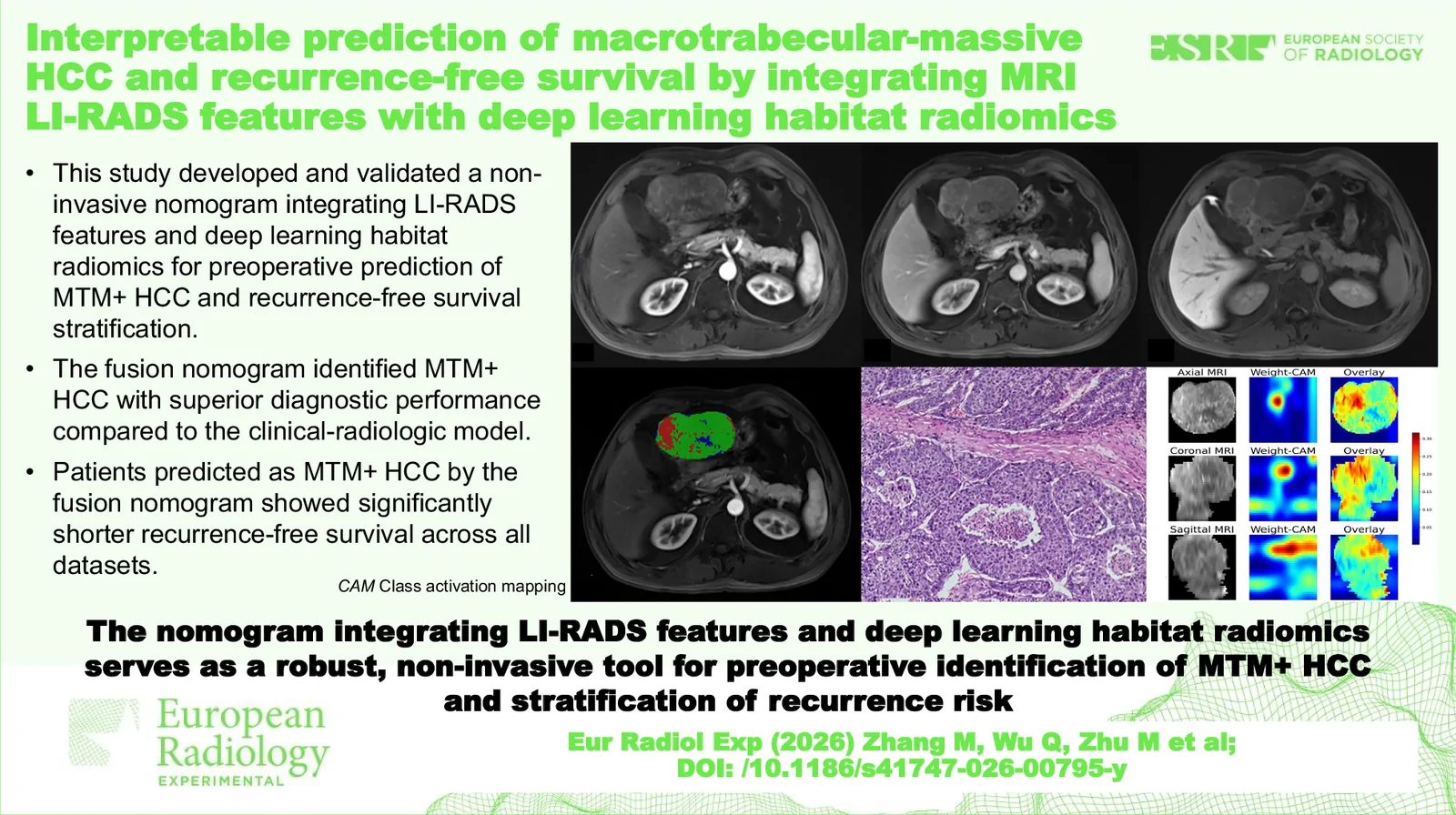

## Background

Primary hepatocellular carcinoma (HCC) is one of the most common malignant tumors of the liver, and has a high recurrence rate after surgical resection [1, 2]. Previous studies have demonstrated that angiogenesis and intratumoral heterogeneity are key features of HCC and can be evaluated noninvasively [3, 4]. Macrotrabecular-massive (MTM + ) HCC, a distinct histological subtype of HCC, has garnered considerable attention due to its association with poor prognosis and early recurrence [5–7]. A recent study has identified Endothelial-Specific Molecule 1 as a potential reliable biomarker for MTM + HCC [8]. However, immunohistochemical detection requires pathological specimens, highlighting the important role of preoperative noninvasive diagnosis.

Several diagnostic models based on radiomics features have demonstrated good performance in predicting MTM + HCC [9, 10]. Traditional radiomics models primarily focused on features extracted from the entire tumor, often overlooking its spatial heterogeneity. In contrast, habitat radiomics analyzes imaging features derived from specific habitats within the tumor, offering more nuanced insights than conventional whole-tumor analysis [11, 12]. Xie et al [13] established a prognostic model using habitat radiomics features derived from specific subregions in esophageal tumors, which achieved superior predictive performance compared to a whole-tumor-based radiomics model. Previous studies have shown that HCC, particularly MTM + HCC, is marked by extensive heterogeneity in its imaging characteristics [14–16]. Given this heterogeneity, habitat radiomics may improve performance in identifying MTM + HCC.

Deep learning (DL) models, which leverage large datasets to select effective imaging features, have been successfully applied for the preoperative prediction of vessels encapsulating tumor clusters and microvascular invasion in HCC [17–19], demonstrating high predictive performance. Li et al [20] developed a DL radiomics model using dual-energy computed tomography (CT), achieving an area under the receiver operating characteristic curve (AUC) of 0.89 (95% confidence interval [CI]: 0.81–0.97) for predicting MTM + HCC in the external test set. To our knowledge, no studies have simultaneously utilized both habitat radiomics and DL features to predict MTM + HCC.

Our study aimed to develop and validate a DL habitat radiomics fusion nomogram based on gadolinium-ethoxybenzyl-diethylenetriamine pentaacetic acid (Gd-EOB-DTPA)-enhanced MRI that integrated clinical, radiologic, habitat radiomics, and DL features to predict MTM + HCC and stratify patients by recurrence‑free survival (RFS) risk.

## Materials and methods

### Ethics approval

This multicenter study was approved by the institutional review boards of all three participating hospitals (Institute 1: the First Affiliated Hospital of Soochow University, No. 2024431; Institute 2: the Nantong Third People’s Hospital, No. EK2023025; Institute 3: the First People’s Hospital of Taicang, No. 2022-ky-203), and informed consent was waived due to its retrospective nature.

#### Patients and clinical data

**Fig. 1:**
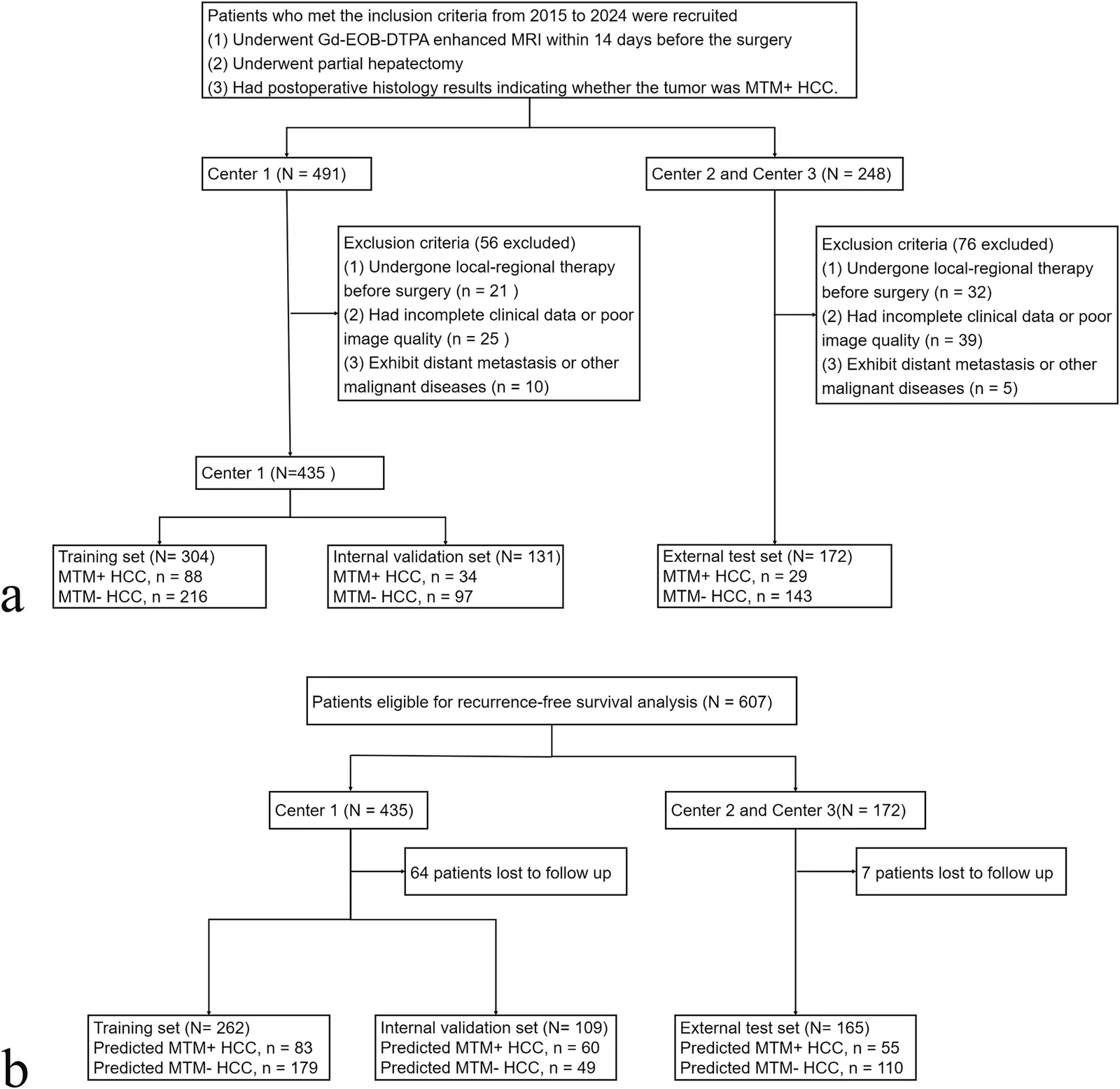
**a** Flowchart of patient selection. **b** Flowchart of patients enrolled for recurrence-free survival analysis, derived from the final cohort in **a**. Gd-EOB-DTPA, Gadolinium-ethoxybenzyl-diethylenetriamine pentaacetic acid, MTM + HCC, Macrotrabecular-massive hepatocellular carcinoma

This retrospective study was approved by the institutional review boards of all participating medical centers, with a waiver of the requirement for informed consent. The study included 739 patients with pathologically confirmed HCC between June 2015 and January 2024 from three centers. Patients were included if they (1) underwent Gd-EOB-DTPA enhanced MRI within 14 days before the surgery; (2) underwent partial hepatectomy; and (3) had postoperative histology results indicating whether the tumor was MTM + HCC. Patients were excluded if they (1) underwent local-regional therapy before surgery; (2) had incomplete clinical data or poor image quality. (3) exhibited distant metastasis or other malignant diseases. Finally, a total of 607 patients with early-stage HCC who underwent curative-intent surgical resection were included; 435 patients from center 1 were randomly assigned to a training set (*n* = 304) and an internal validation set (*n* = 131) with a ratio of 7:3, and patients from centers 2 and 3 (*n* = 172) were used as the external test set. The flowchart of patient recruitment is shown in Fig. 1. If multiple lesions were present in the liver, the largest lesion was selected for the study.

Clinical and laboratory data for all patients were retrospectively collected from electronic medical records, including sex, age, viral hepatitis status, cirrhosis, tumor number, maximum tumor diameter, and levels of alpha-fetoprotein (AFP), aspartate transaminase (AST), alanine transferase (ALT), and γ-glutamyl transferase (GGT).

#### Histopathology analysis

All histologic slides were independently reviewed by two senior pathologists (C.G. and M.D., both with 8 years of experience). According to the 2019 World Health Organization classification [5], MTM structure was defined as the presence of tumor cell layers exceeding 6 layers. When more than 50% of the tumor volume exhibited MTM structure, it was classified as MTM + HCC, and those with less than 50% MTM structure were categorized as MTM- HCC. The interobserver agreement for MTM classification was excellent (κ = 0.92) and was assessed in the entire cohort of 607 patients from all three centers.

#### MRI acquisition

MRI was performed using three 3-T systems: MAGNETOM Verio (Siemens Healthcare, Erlangen, Germany), Achieva (Philips Medical Systems, Best, The Netherlands), and Discovery MR750 (GE Healthcare, Chicago, IL, USA), each equipped with 16-channel phased array torso coils. Gd-EOB-DTPA (Primovist; Bayer Schering Pharma, Leverkusen, Germany) was injected using a high-pressure syringe at a flow rate of 1–2 mL/s, with a total dose of 0.025 mmol/kg. Dynamic phases were acquired after contrast injection using voxel-interpolated breath-hold fat-suppressed T1-weighted imaging. Arterial phase (AP), portal venous phase (PP), and hepatobiliary phase (HBP) images were obtained at 30 s, 60 s, and 20 min after contrast injection, respectively. Specific parameters for the different scanners are detailed in Supplementary Table S1.

#### MRI feature analysis

All MRI data were retrospectively analyzed by two radiologists (M.Z. and Q.W., with 3 and 5 years of experience, respectively). The following qualitative imaging features were evaluated primarily based on Liver Imaging Reporting and Data System (LI-RADS) and previous studies [21–23]: infiltrative appearance, fat in mass, blood product in mass, enhancement pattern (nonrim arterial phase hyperenhancement [APHE] and rim APHE), washout pattern (nonperipheral washout and peripheral washout), type of capsule (enhancing capsule and non-enhancing capsule), AP peritumoural enhancement, intratumoural artery, substantial necrosis (> 20%, > 50%), satellite nodules, central scar, peritumoral hypointensity in the HBP, gadolinium-enhanced clouding, LI-RADS category. Inter-reader agreement for the above LI-RADS-related imaging features and LI-RADS category, assessed in the entire cohort of 607 patients from all three centers, was excellent (κ range: 0.86–0.91). Details of features are described in Supplementary Material Appendix S1.

#### Automatic segmentation using nnU‑Net

Three-dimensional volume-of-interest regions of 353 HCC lesions in three data sets were manually contoured on AP, PP, and HBP images by two junior radiologists (M.Z. and Q.W., with 3 and 5 years of experience) in consensus using 3D-slicer 5.6.2 (https://www.slicer.org/). The nnU-Net framework (https://github.com/MIC-DKFZ/nnUNet) was employed for automatic segmentation. The 353 HCC lesions were randomly split into training (*n* = 282) and validation sets (*n* = 71). The model was trained using the default three-dimensional full-resolution configuration of nnU-Net. Detailed procedures and performance metrics are provided in the Supplementary material (Appendix S2 and Fig. S1). The remaining 254 lesions underwent automatic segmentation using the nnU-Net framework. A senior radiologist (Y.Y., with 12 years of experience) reviewed both the manual segmentations from the two junior radiologists and the model-predicted masks, manually correcting any inaccurate masks to establish the ground truth segmentations. Ultimately, 607 ground truth segmentations verified by the senior radiologist were used for subsequent analyses.

#### Habitat radiomics feature extraction

K-means clustering (*k* = 2–8) was applied to radiomic features, with the Calinski–Harabasz index used to evaluate cluster validity. Based on the highest Calinski–Harabasz index, the optimal number of clusters was determined to be 3 (Supplementary Fig. S2). The derived cluster centroids were then applied unchanged to the internal validation and external test sets to generate three habitat subregion maps, serving as imaging biomarkers for quantifying intratumor heterogeneity. Habitat radiomics features were extracted using PyRadiomics software version 3.0.1 (http://www.radiomics.io/pyradiomics.html). Features were derived from the original, Laplacian-of-Gaussian (with sigma values ranging from 2.0 to 5.0 mm), and wavelet-transformed (eight directional decompositions) images. The extracted feature classes included shape, first-order statistics, gray-level co-occurrence matrix–GLCM, gray-level run-length matrix–GLRLM, gray-level size zone matrix–GLSZM, and gray-level dependence matrix–GLDM. Before feature extraction, all images were resampled to an isotropic voxel spacing of 1 × 1 × 1 mm³ to ensure scale invariance. To compute texture features, image intensities were discretized using a fixed bin width of 5. For each cluster, 1,132 habitat radiomics features were extracted, totaling 3,396 features for a single phase per patient.

#### DL feature extraction

**Fig. 2:**
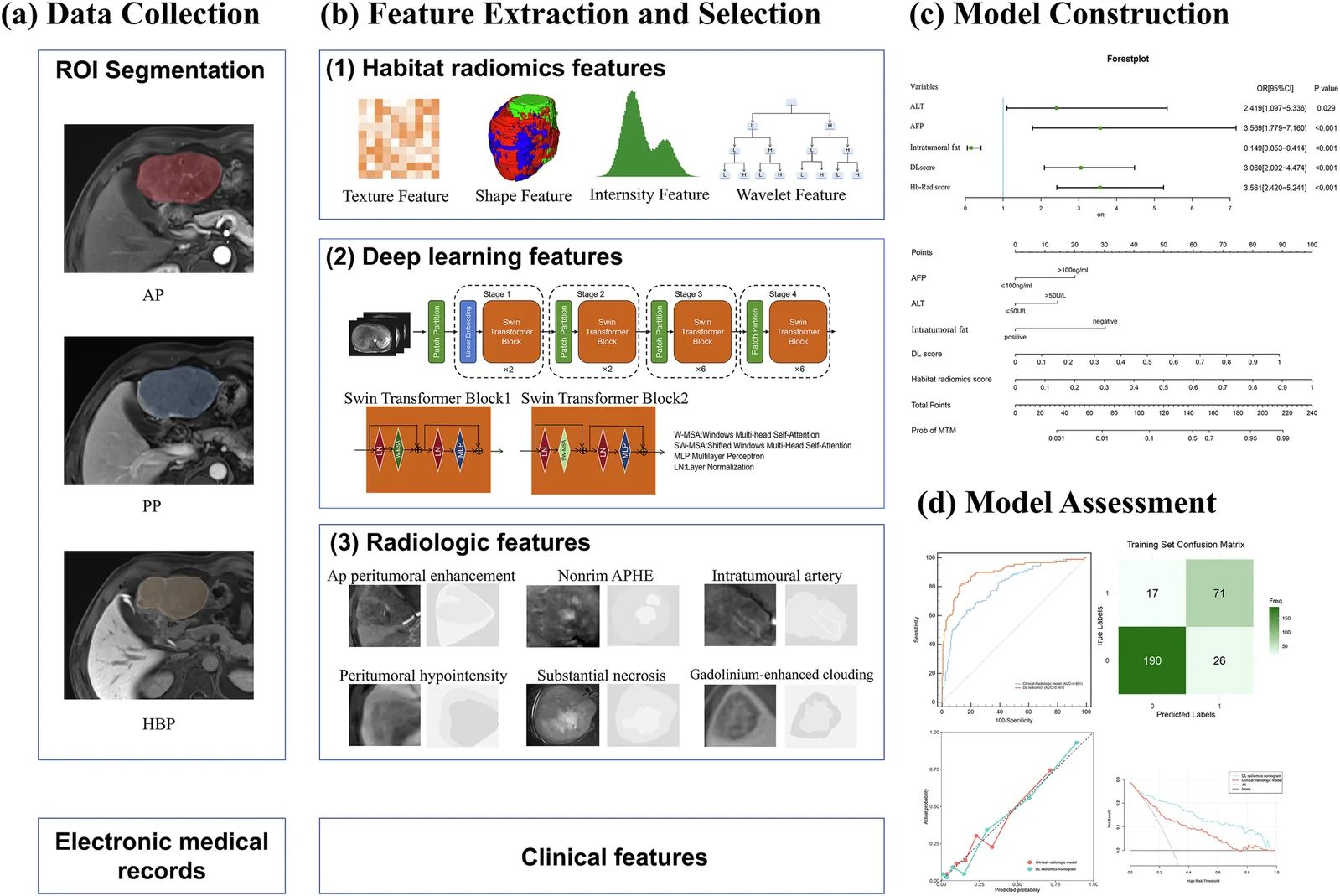
Workflow of the study, including data collection (**a**), feature extraction and selection (**b**), model construction (**c**), and model assessment (**d**). AFP, Alpha-fetoprotein; ALT, Alanine aminotransferase; DL, Deep learning; MTM, Macrotrabecular-massive; VOI, Volume-of-interest

A pretrained (Kinetics dataset-based) 3D Swin Transformer model from the “torchvision” library of the DL framework PyTorch v2.5.1 (https://github.com/pytorch/pytorch) was used to develop our DL models using transfer learning. The code for the 3D Swin Transformer model is available on GitHub (https://github.com/doctoryuxing/MTM-HCC-Swin3d-classification.git). Before model input, all images underwent intensity normalization to scale pixel values to the [0,1] range to reduce scanner-related variations. Additionally, the Swin Transformer architecture incorporates Layer Normalization, which provides feature-wise normalization during training and enhances robustness to input variations. To prevent overfitting during training, we implemented several proven techniques, including advanced data augmentation and scheduled learning rate decay. The augmentation pipeline included transformations such as Resized, ScaleIntensityRanged, RandFlipd, and RandAffined. We used a 16-batch size and an initial learning rate of 0.0001, with the learning rate decaying by a factor of 0.1 every 7 epochs. To handle class imbalance, we applied focal loss and the Adam optimizer, which was selected for its automatic learning rate adjustments. The training process lasted between 200 and 500 epochs. After completion of training, the final model weights were saved and used for subsequent deep feature extraction. The training and validation learning curves of the AP are provided in Supplementary Fig. S3, demonstrating stable convergence without overfitting. The process is presented in Fig. 2.

#### Construction of models

In the training set, univariable analysis and multivariable stepwise logistic regression analysis with Akaike information criterion were used to determine the clinical and radiologic features to include in the clinical-radiologic (CR) model.

Habitat radiomics features from three phases (AP, PP, and HBP) were integrated to generate the habitat radiomics combined-phase (CP) features. Similarly, DL features extracted from the same three phases were fused to create the DL CP features. Habitat radiomics and DL features were processed and selected by FeAture Explorer V 0.5.12 (https://github.com/salan668/FAE). The detailed procedure is listed in the Appendix S3. After feature normalization and selection, habitat radiomics models were built with the support-vector-machine (SVM) classifier, whereas DL models were constructed using the autoencoder classifier. The best-performing habitat radiomics and DL models were selected according to their respective internal validation set AUCs. Habitat radiomics score and DL score for MTM + HCC were calculated based on the mean predicted probabilities from their respective best-performing models.

To enhance the interpretability of the DL models, we generated class activation maps to visualize the key regions identified by the models. The class activation map utilized color mapping to display the importance of different tumor regions in the classification task.

#### DL habitat radiomics fusion nomogram construction

All variables with *p* < 0.05 (including clinical, radiologic, habitat radiomics score, and DL score) were entered into a multivariable backward logistic regression to identify independent predictors of MTM + HCC. Finally, all selected variables were used to build the DL habitat radiomics fusion model using the Logistic regression method. The DL habitat radiomics fusion nomogram was generated for visualization of the model. Calibration curves and confusion matrices were used to evaluate the performance of the nomogram. Decision curve analysis was applied to assess the clinical benefits of the nomogram. Model interpretability was further analyzed using Shapley additive explanations (SHAP).

#### Cox regression analysis for RFS

Patients underwent postoperative clinical and radiologic follow-up at intervals of 3–6 months (Fig. 1). RFS was calculated from the date of surgery to the radiographic detection of tumor recurrence or death, or until the last follow-up if no recurrence was observed. Follow-up continued until December 30, 2024.

On the basis of the training set, clinical features and nomogram-predicted MTM state (predicted MTM+ and predicted MTM−) were entered into univariable and multivariable Cox regression analysis. Independent predictors (*p* < 0.05) were then integrated to construct a prognostic model for RFS. The model performance was assessed by the *C*-index.

#### Statistical analysis

Statistical analyses were conducted using SPSS 26.0, R software 4.4.1, and Python 3.12. Categoric variables are expressed as numbers and percentages, continuous variables are expressed as the mean ± standard deviation or median with interquartile range. The Chi-Square test was used to compare frequencies of categorical variables between MTM+ HCCs and MTM- HCCs. The Kolmogorov–Smirnov test was used to assess the normality of continuous variables. The independent-samples *t*-test or the Mann–Whitney *U*-test was performed for continuous variables. Spearman analysis was used to evaluate the correlation between features. The AUCs of different models were compared by the DeLong test. Kaplan–Meier analysis with the log-rank test was used to compare the prognosis rate. For the pairwise comparisons among the habitat radiomics models and DL models, the Bonferroni correction was applied by adjusting the significance threshold to *p* < 0.0083 to control the family-wise error rate. For all other analyses, a two-tailed *p* < 0.05 was considered indicative of a statistically significant difference. Receiver operating characteristic curves were drawn with MedCalc 20.022 (https://www.medcalc.org/). R packages including “survival”, “survminer” were used for survival analysis and visualization. The “shap” in Python was used to generate SHAP of the model.

## Results

### Clinical and radiologic characteristics of patients

The clinical and radiologic characteristics of patients are presented in Tables 1 and  2. A total of 607 patients (474 males and 133 females; aged 59.6 ± 10.6 years [mean ± standard deviation]) were included in this study, with 151 MTM + HCC (122 from center 1, 29 from centers 2 and 3). In the training set, patients diagnosed with MTM + HCC were younger and exhibited larger maximum tumor diameters compared to MTM- HCC patients (*p* = 0.028 and < 0.001, respectively). Among other variables, ALT (*p* = 0.008), AST (*p* < 0.001), GGT (*p* = 0.013), and AFP (*p* < 0.001) were found to be significantly higher in the MTM+ group than in the MTM- HCC group. There were no significant differences in the remaining baseline characteristics between the two groups.

**Table 1:** Patient characteristics in the training, internal validation, and external test sets

Characteristics	Training set (*n* = 304)				Internal validation set (*n* = 131)	External test set (*n* = 172)	*p*-value^a^	*p*-value^b^
	Total	Positive MTM (*n* = 88)	Negative MTM (*n* = 216)	*p*-value				
Sex				0.100			0.462	0.120
Male	237 (78.0)	74 (84.1)	163 (75.5)		108 (82.4)	129 (75.0)		
Female	67 (22.0)	14 (15.9)	53 (24.5)		23 (17.6)	43 (25.0)		
Age	60.05 ± 11.21	57.84 ± 10.12	60.94 ± 11.53	0.028	60.13 ± 10.24	58.49 ± 9.65	0.113	0.301
Viral hepatitis				0.556			< 0.001	0.019
No	82 (27.0)	20 (22.7)	62 (28.7)		29 (22.1)	20 (11.6)		
HBV	212 (69.7)	66 (75.0)	146 (67.6)		98 (74.8)	151 (87.8)		
HCV	7 (2.3)	1 (1.1)	6 (2.8)		2 (1.5)	1 (0.6)		
HBV + HCV co-infection	3 (1.0)	1 (1.1)	2 (0.9)		2 (1.5)	0 (0.0)		
Cirrhosis				0.156			< 0.001	0.048
Yes	143 (47.0)	47 (53.4)	96 (44.4)		69 (52.7)	110 (64.0)		
No	161 (53.0)	41 (46.6)	120 (55.6)		62 (47.3)	62 (36.0)		
ALT(%)				0.008			0.069	0.381
> 50 U/L	61 (20.1)	26 (29.5)	35 (16.2)		30 (22.9)	47 (27.3)		
≤ 50 U/L	243 (79.9)	62 (70.5)	181 (83.8)		101 (77.1)	125 (72.7)		
AST(%)				< 0.001			0.010	0.100
> 40 U/L	90 (29.6)	39 (44.3)	51 (23.6)		42 (32.1)	71 (41.3)		
≤ 40 U/L	214 (70.4)	49 (55.7)	165 (76.4)		89 (67.9)	101 (58.7)		
GGT (%)				0.013			0.933	0.220
> 60 U/L	119 (39.1)	44 (50.0)	75 (34.7)		61 (46.6)	68 (39.5)		
≤ 60 U/L	185 (60.9)	44 (50.0)	141 (65.3)		70 (53.4)	104 (60.5)		
AFP(%)				< 0.001			0.057	0.262
> 100 ng/mL	124 (40.8)	53 (60.2)	71 (32.9)		50 (38.2)	55 (32.0)		
≤ 100 ng/mL	180 (59.2)	35 (39.8)	145 (67.1)		81 (61.8)	117 (68.0)		
Tumor number				0.590			0.013	0.088
≥ 2	90 (29.6)	28 (31.8)	62 (28.7)		36 (27.5)	33 (19.2)		
1	214 (70.4)	60 (68.2)	154 (71.3)		95 (72.5)	139 (80.8)		
Maximum diameter (cm)	4.10 (2.50, 7.18)	6.30 (3.52, 9.78)	3.60 (2.40, 5.70)	< 0.001	3.90 (2.50,6.30)	3.00 (1.90, 4.58)	< 0.001	0.001

Data are numbers of patients, with percentages in parentheses

*AFP* Alpha-fetoprotein, *ALT* Alanine aminotransferase, *AST A*spartate aminotransferase, *GGT* Gamma-glutamyl transferase, *MTM* + *HCC* Macrotrabecular-massive hepatocellular carcinoma

*p*-value < 0.05 indicates a significant difference between MTM + HCC and MTM- HCC in the training set

^a^ *p*-value < 0.05 indicates a significant difference between the training and external test set

^b^ *p*-value < 0.05 indicates a significant difference between the internal validation and external test set

**Table 2:** Radiologic characteristics in the training, internal validation, and external test sets

Characteristics	Training set (*n* = 304)				Internal validation set (*n* = 131)	External test set (*n* = 172)	*p*-value^a^	*p*-value^b^
	Total	MTM + HCC (*n* = 88)	MTM- HCC (*n* = 216)	*p*-value				
Infiltrative appearance	99 (32.6)	41 (46.6)	58 (26.9)	0.001	46 (35.1)	60 (34.9)	0.606	0.967
Blood product in mass	66 (21.8)	31 (35.2)	35 (16.3)	< 0.001	27 (20.6)	24 (14.0)	0.036	0.125
Fat in mass	71 (23.4)	9 (10.2)	62 (28.7)	0.001	21 (16.0)	36 (20.9)	0.543	0.280
Enhancement pattern				0.066			0.508	0.330
Nonrim APHE	255 (83.9)	71 (80.7)	184 (85.2)		109 (83.2)	137 (79.7)		
Rim APHE	31 (10.2)	14 (15.9)	17 (7.9)		10 (7.6)	22 (12.8)		
Washout				0.017			0.061	0.005
Nonperipheral washout	208 (68.4)	61 (69.3)	147 (68.1)		83 (63.4)	128 (74.4)		
Peripheral washout	18 (5.9)	10 (11.4)	8 (3.7)		6 (4.6)	15 (8.7)		
Capsule				0.048			0.009	0.548
Non-enhancing capsule	41 (13.5)	15 (17.0)	26 (12.0)		10 (7.6)	8 (4.7)		
Enhancing capsule	137 (45.1)	46 (52.3)	91 (42.1)		63 (48.1)	84 (48.8)		
AP peritumoural enhancement	44 (14.5)	15 (17.0)	29 (13.4)	0.416	24 (18.3)	19 (11.0)	0.289	0.072
Intratumoural artery	118 (38.8)	50 (56.8)	68 (31.5)	< 0.001	43 (32.8)	42 (24.4)	0.001	0.107
Substantial necrosis				< 0.001			0.073	0.902
> 20%	91 (29.9)	34 (38.6)	57 (26.4)		31 (23.7)	37 (21.5)		
> 50%	29 (9.5)	15 (17.0)	14 (6.5)		10 (7.6)	13 (7.6)		
Satellite nodules	40 (13.2)	16 (18.2)	24 (11.1)	0.098	22 (16.8)	6 (3.5)	0.001	< 0.001
Central scar	44 (14.5)	19 (21.6)	25 (11.6)	0.024	17 (13.0)	31 (18.0)	0.307	0.233
Peritumoural hypointensity in the HBP	45 (14.8)	24 (27.3)	21 (9.7)	< 0.001	21 (16.0)	17 (9.9)	0.126	0.109
Gadolinium-enhanced clouding	18 (5.9)	4 (4.5)	14 (6.5)	0.517	9 (6.9)	19 (11.0)	0.045	0.214
LI-RADS				0.022			0.101	0.408
LR-3	20 (6.6)	2 (2.3)	18 (8.3)		12 (9.2)	19 (11.0)		
LR-4	46 (15.1)	9 (10.2)	37 (17.1)		19 (14.5)	16 (9.3)		
LR-5	207 (68.1)	63 (71.6)	144 (66.7)		86 (65.6)	123 (71.5)		
M	31 (10.2)	14 (15.9)	17 (7.9)		14 (10.7)	14 (8.1)		

*AP* Arterial phase, *APHE* Arterial phase hyperenhancement, *HBP* Hepatobiliary phase, *MTM* + *HCC* Macrotrabecular-massive hepatocellular carcinoma

*p*-value < 0.05 indicates a significant difference between MTM + HCC and MTM- HCC in the training set

^a^ *p*-value < 0.05 indicates a significant difference between the training and external test set

^b^ *p*-value < 0.05 indicates a significant difference between the internal validation and external test set

After univariable analysis, infiltrative appearance (*p* = 0.001), blood product in mass (*p* < 0.001), fat in mass (*p* = 0.001), washout pattern (*p* = 0.017), type of capsule (*p* = 0.048), intratumoural artery (*p* < 0.001), substantial necrosis (*p* < 0.001), central scar (*p* = 0.024), peritumoral hypointensity in the HBP (*p* < 0.001), and LI-RADS category (*p* = 0.022) were found to be associated with MTM + HCC.

#### Segmentation performance of nnU-Net

To evaluate segmentation accuracy, we compared the nnU-Net-generated masks (*n* = 254) with the ground truth segmentations verified by the senior radiologist (Y.Y., with 12 years of experience). In addition to the Dice similarity coefficient, we calculated the 95th percentile Hausdorff distance to assess boundary precision. Overall, Dice similarity coefficients were 0.891 for AP, 0.883 for PP, and 0.932 for HBP, while 95th percentile Hausdorff distance values were 13.4 mm for AP, 13.2 mm for PP, and 10.4 mm for HBP. To further evaluate robustness across different tumor sizes, we stratified the performance by lesion size (≤ 3 cm, 3–5 cm, and > 5 cm). The results are summarized in Supplementary Table S2.

### Performance of models

Based on multivariable logistic regression, ALT (odds ratio [OR] = 2.435 [95% CI: 1.242–4.772], *p* = 0.010), AFP (OR = 2.839 [95% CI: 1.584–5.085], *p* < 0.001), maximum tumor diameter (OR = 1.782 [95% CI: 1.329–2.389], *p* < 0.001), fat in mass (OR = 0.159 [95% CI: 0.065–0.389], *p* < 0.001), type of capsule (enhancing capsule: OR = 1.782 [95% CI: 0.944–3.366], *p* = 0.075; nonenhancing capsule: OR = 2.343 [95% CI: 0.953–5.759], *p* = 0.064) (no capsule as reference) and peritumoral hypointensity in the HBP (OR = 2.675 [95% CI: 1.234–5.799], *p* = 0.013) were selected for CR model construction (Supplementary Table S3). The CR model had AUCs of 0.801 (95% CI: 0.746–0.856) in the training set, 0.651 (95% CI: 0.563–0.732) in the internal validation set, and 0.739 (95% CI: 0.666–0.803) in the external test set.

Finally, we established four habitat radiomics models and four DL models based on AP, PP, HBP, and CP features. Among all habitat radiomics models, the CP model achieved the best performance, with AUCs of 0.802 (95% CI: 0.747–0.858) in the training set, 0.687 (95% CI: 0.585–0.789) in the internal validation set, and 0.681 (95% CI: 0.553–0.808) in the external test set. The CP DL model demonstrated the highest AUC among all DL models, achieving AUCs of 0.740 (95% CI: 0.676–0.804), 0.712 (95% CI: 0.618–0.805), and 0.725 (95% CI: 0.624–0.825) in the training, internal validation, and external test sets, respectively. All habitat radiomics models and DL models were evaluated for diagnostic performance and comparison. The results are shown in Supplementary Appendix S4, Tables S4–S6, and Fig. S4. The correlations between the features of two CP models are presented in Supplementary Fig. S5.

In the training, internal validation, and external test sets, median DL scores for the MTM + HCC group were 0.569, 0.478, and 0.518, respectively—significantly higher than those of the MTM − HCC group (0.377, 0.415, and 0.369; *p* < 0.001 for all sets). Similarly, median habitat radiomics scores for MTM + HCC were 0.591, 0.492, and 0.373 in the training, internal validation, and external test sets, *versus* 0.334, 0.362, and 0.266 for MTM − HCC (training set: *p* < 0.001; internal validation set: *p* = 0.001; external test set: *p* = 0.002). Comparisons of DL and habitat radiomics scores between MTM+ and MTM − HCC groups are presented in Supplementary Table S7.

**Fig. 3:**
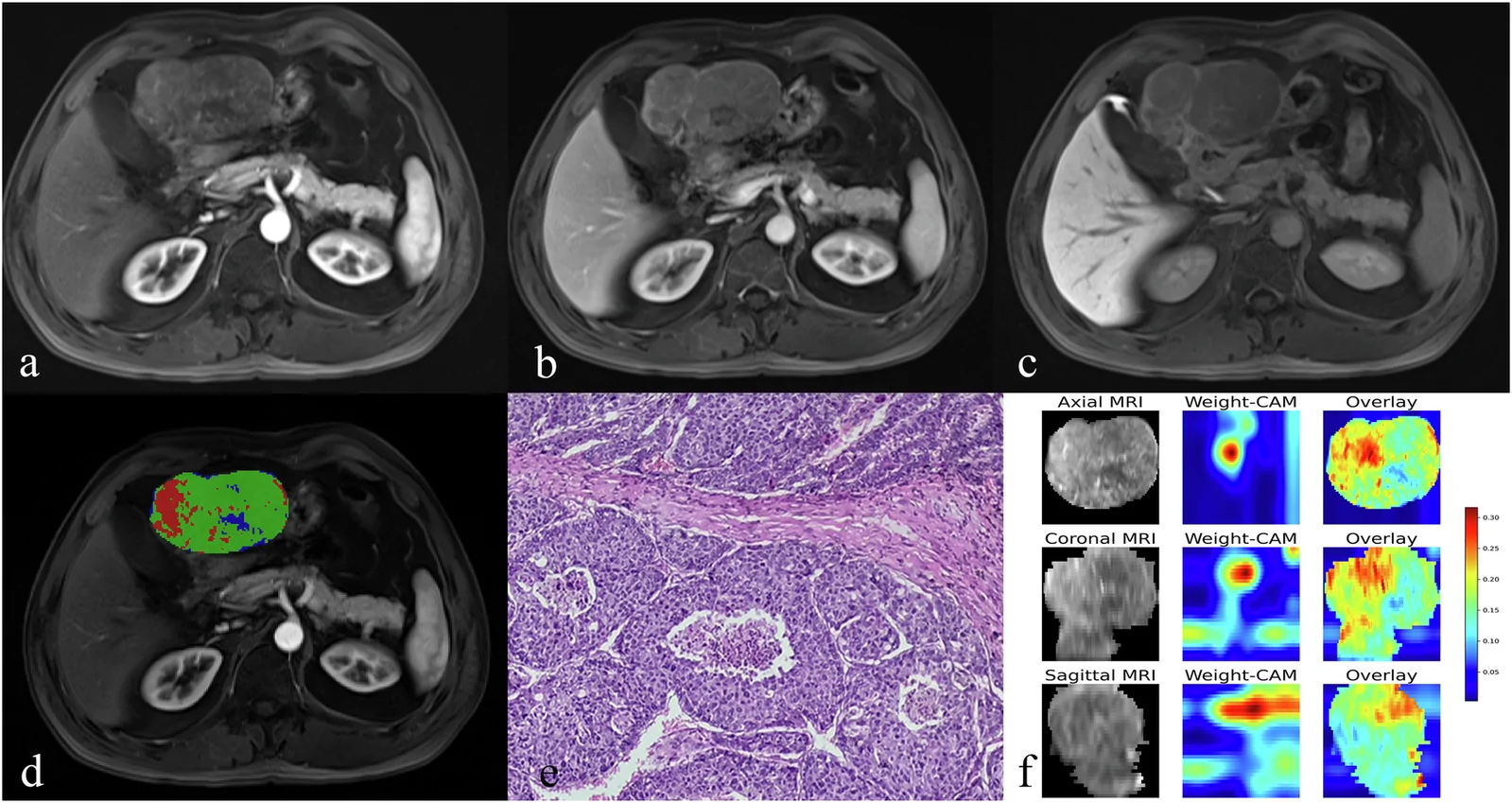
**a** AP, (**b**) PP, and (**c**) HBP MRI images showing a tumor located in the left liver lobe of a 54-year-old male patient with macrotrabecular-massive hepatocellular carcinoma (MTM + HCC). **d** Volume of Interest delineated during the AP. Different colors in the figure represent different sub-regions. The red area corresponds to the markedly enhanced portion of the tumor, while the blue area indicates a small amount of necrosis within the tumor. The green area is distributed within the tumor dense cellular areas. **e** Hematoxylin-eosin staining (original magnification, ×100) of MTM + HCC reveals the macro-trabecular massive pattern. **f** The Weighted Class Activation Mapping heatmaps for the Swin Transformer model in AP. The red regions indicate areas of activation associated with MTM + HCC

In the CAM of the Swin Transformer (Fig. 3), the red areas highlighted the regions that the model prioritized most during the prediction task.

### Performance and Interpretability of the nomogram

**Fig. 4:**
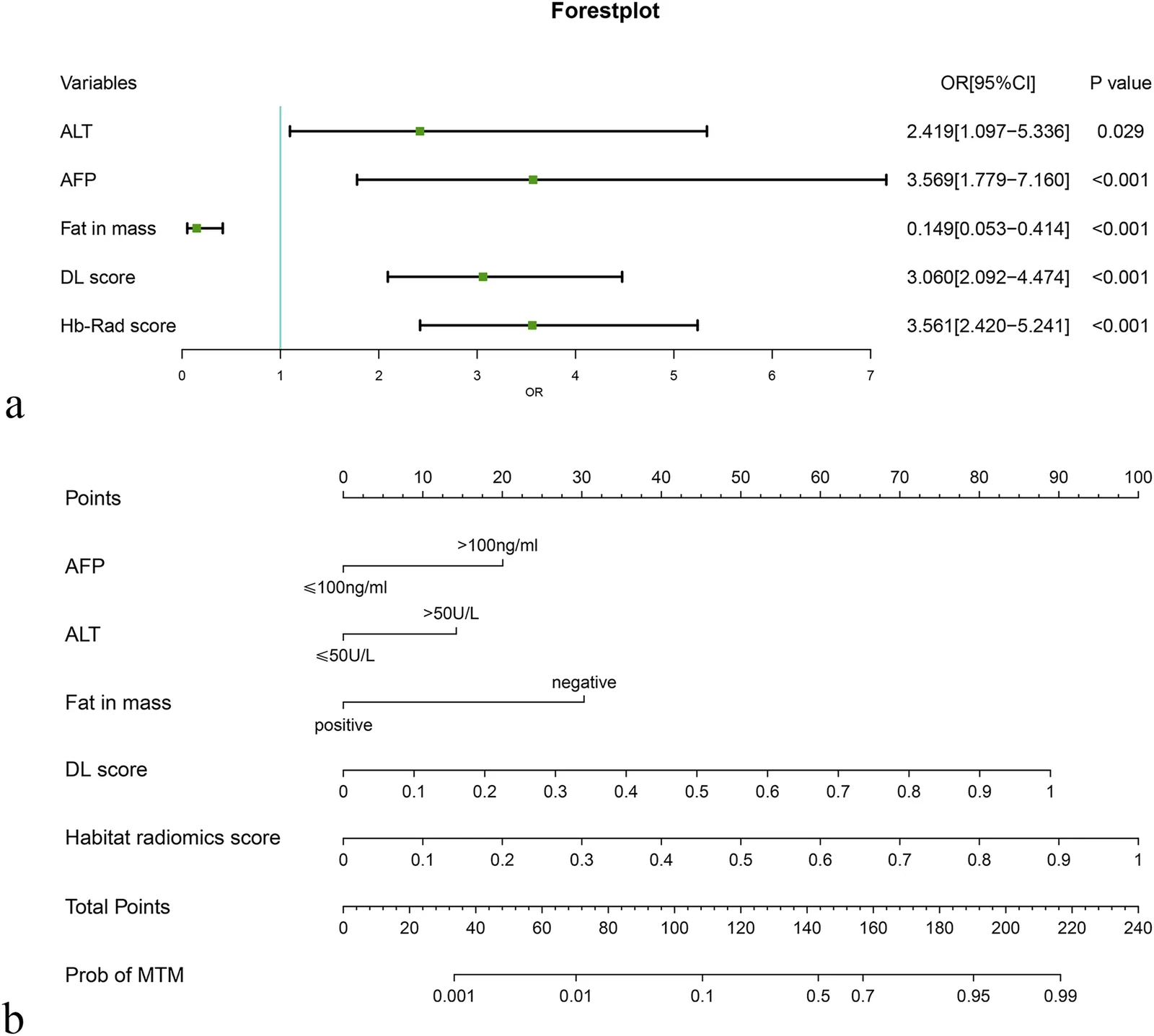
**a** Forest plot for identifying MTM + HCC in the DL habitat radiomics fusion nomogram. **b** The DL habitat radiomics fusion nomogram for identifying MTM + HCC preoperatively. AFP, Alpha-fetoprotein; ALT, Alanine aminotransferase; DL, Deep learning; Hb-Rad, Habitat radiomics; MTM, Macrotrabecular-massive

After multivariable analysis, habitat radiomics score (OR = 3.561 [95% CI: 2.420–5.241], *p* < 0.001), DL score (OR = 3.060 [95% CI: 2.092–4.474], *p* < 0.001), AFP (OR = 3.569 [95% CI: 1.779–7.160], *p* < 0.001), ALT (OR = 2.419 [95% CI: 1.097–5.336], *p* = 0.029), and fat in mass (OR = 0.149 [95% CI: 0.053–0.414], *p* < 0.001) were independent predictors of MTM + HCC (Supplementary Table S8 and Fig. 4a). The correlation between the features of the DL habitat radiomics fusion nomogram was presented in Supplementary Fig. S6.

The nomogram (Fig. 4b) showed excellent performance with AUCs of 0.897 (95% CI: 0.854–0.940), 0.757 (95% CI: 0.674–0.827), and 0.858 (95% CI: 0.797–0.906) in the training, internal validation, and external test sets, respectively (Table 3).

**Table 3:** Comparison of CR model and DL habitat radiomics fusion nomogram for predicting MTM + HCC

Variables	AUC [95% CI]	Brier score	ECE	Calibration slope	F1 score [95% CI]	sensitivity [95% CI]	specificity [95%CI]	Accuracy	*z*-value	*p*-value
Training set										
CR model	0.801 [0.746–0.856]	0.154	0.047	1.885	0.619 [0.535–0.696]	0.636 [0.532–0.734]	0.829 [0.776–0.878]	0.773	4.211	< 0.0001
DL habitat radiomics fusion nomogram	0.897 [0.854–0.940]	0.105	0.045	0.989	0.768 [0.698–0.830]	0.807 [0.718–0.888]	0.880 [0.834–0.919]	0.859		
Internal validation set										
CR model	0.651 [0.563–0.732]	0.200	0.128	0.536	0.504 [0.383–0.606]	0.882 [0.764–0.977]	0.433 [0.333–0.528]	0.550	2.383	0.0172
DL habitat radiomics fusion nomogram	0.757 [0.674–0.827]	0.177	0.100	0.413	0.566 [0.449–0.667]	0.882 [0.769–0.974]	0.567 [0.468–0.663]	0.649		
External test set										
CR model	0.739 [0.666–0.803]	0.127	0.076	1.543	0.425 [0.281–0.548]	0.586 [0.393–0.769]	0.762 [0.692–0.827]	0.732	2.430	0.0151
DL habitat radiomics fusion nomogram	0.858 [0.797–0.906]	0.093	0.059	1.037	0.550 [0.413–0.667]	0.862 [0.684–0.960]	0.741 [0.683–0.821]	0.762		

*AUC* Area under the receiver operating characteristic curve, *CI* Confidence interval, *CR* Clinical-radiologic model, *DL* Deep learning, *ECE* Expected calibration error, *MTM* + *HCC* Macrotrabecular-massive hepatocellular carcinoma

**Fig. 5:**
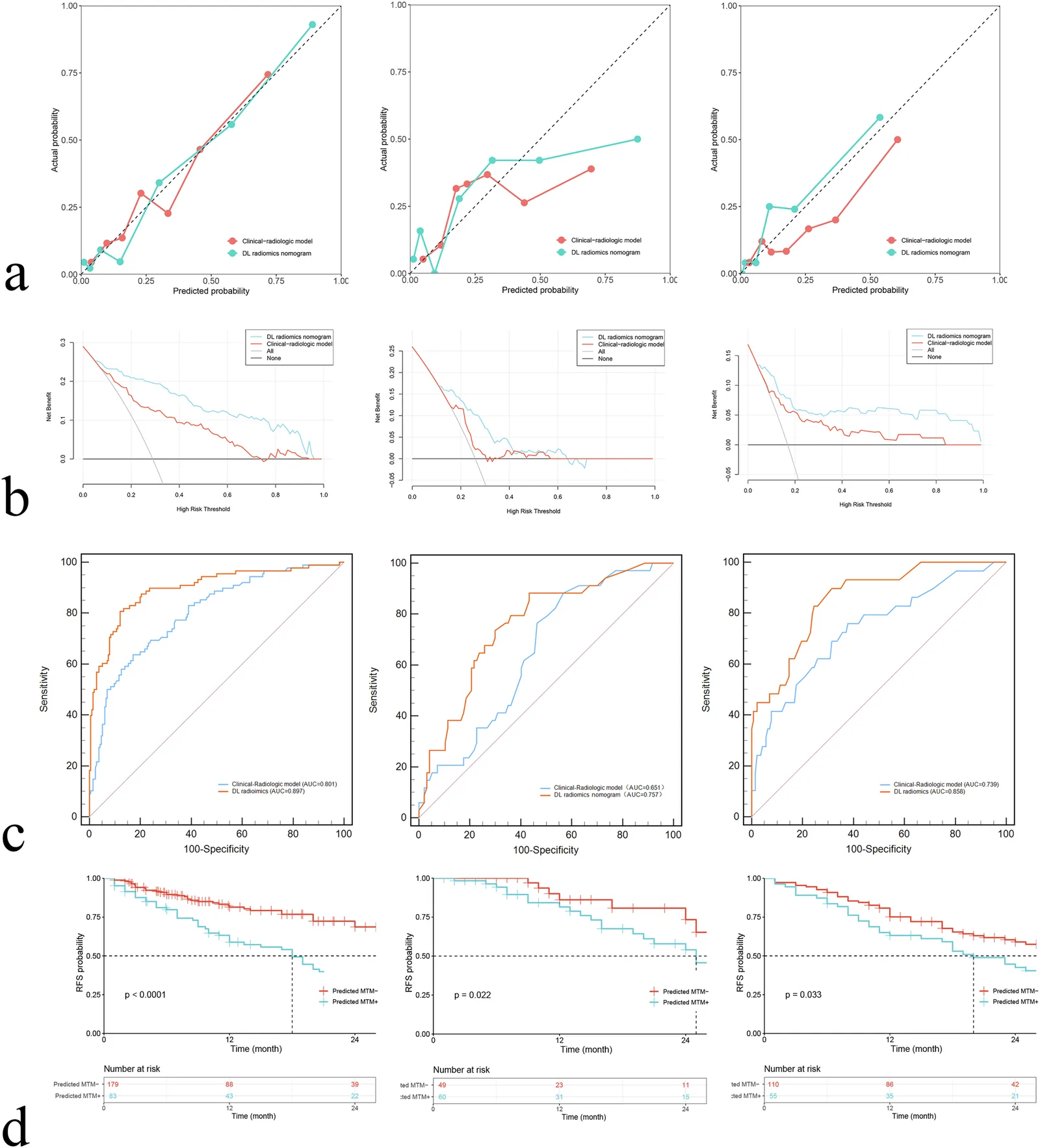
Calibration curves (**a**), decision curve analysis (**b**), and receiver operating characteristic curves (**c**) of the DL habitat radiomics fusion nomogram and the CR model in the training, internal validation, and external test sets. Recurrence-free survival curves (**d**) for the predicted MTM+ and predicted MTM- patients identified by the DL habitat radiomics fusion nomogram in the training, internal validation, and external test sets. AUC, Area under the receiver operating characteristic curve; DL, Deep learning; MTM, Macrotrabecular-massive

As shown in Fig. 5a, the calibration curve indicated that the nomogram had good diagnostic ability across three datasets. Decision curve analysis showed that the nomogram had good clinical utility in identifying MTM + HCC (Fig. 5b). To further analyze the performance of the nomogram, we constructed a confusion matrix based on the predictions (Supplementary Fig. S7).

There is a statistically significant improvement of the performance between the AUC of the nomogram and the CR model in the training (0.801 [95% CI: 0.746–0.856] *versus* 0.897 [95% CI: 0.854–0.940], *p* < 0.001), internal validation (0.651 [95% CI: 0.563–0.732] *versus* 0.757 [95% CI: 0.674–0.827], *p* = 0.017), and external test sets (0.739 [95% CI: 0.666–0.803] *versus* 0.858 [95% CI: 0.797–0.906], *p* = 0.015) (Table 3 and Fig. 5c).

SHAP analysis (Supplementary Fig. S8) revealed positive contributions from habitat radiomics score, DL score, AFP, and ALT, and a negative contribution from fat mass to the prediction of MTM + HCC.

### Cox regression analysis for RFS

As of December 30, 2024, 536 of 607 patients (88.3%) were included for RFS analysis, with 71 patients lost to follow-up. Across all centers, recurrence occurred in 231 cases (43.1%). Patients with p&&redicted MTM + HCC demonstrated significantly poorer RFS (training set: 18.9 months (95% CI: 15.8–22.0), internal validation set: 26.3 months (95% CI: 20.3–32.3), external test set: 30.0 months (95% CI: 22.8–37.1) compared to those with predicted MTM- HCC (training set: 39.3 months (95% CI: 31.7–46.9), internal validation set: 40.5 months (95% CI: 27.2–53.9), external test set: 46.4 months (95% CI: 36.2–56.6)) across all sets (training set: *p* < 0.001, internal validation set: *p* = 0.022, external test set: *p* = 0.033). Kaplan–Meier curves of RFS are shown in Fig. 5d. As detailed in Supplementary Table S9, the type and method of surgery were comparable across all study groups, with no significant differences observed in any of the three comparisons (MTM + HCC *versus* MTM- HCC: *p* = 0.546, 0.152; training set *versus* external test set: *p* = 0.131, 0.253; internal validation set *versus* external test set: *p* = 0.796, 0.338).

In univariable analysis, AFP, ALT, AST, diameter, GGT and nomogram-predicted state were associated with RFS (Table 4). After multivariable analysis, AFP (HR = 2.76, 95% CI: 1.74–4.36, *p* < 0.001) and nomogram-predicted MTM state (HR = 1.72, 95% CI: 1.03–2.88, *p* = 0.039) were identified as independent prognostic factors and used to develop the model for RFS. The model achieved *C*-indexes of 0.74 (95% CI: 0.67–0.81), 0.62 (95% CI: 0.49–0.76), and 0.63 (95% CI: 0.55–0.71) in the training, internal validation, and external test sets.

**Table 4:** Univariable and multivariable Cox regression analysis for RFS in the training set

Characteristics	Univariable analysis			Multivariable analysis		
	HR	95% CI	*p*-value	HR	95% CI	*p*-value
AFP	3.84	2.54–5.82	< 0.001	2.76	1.74–4.36	< 0.001
ALT	1.72	1.13–2.63	0.012	1.06	0.63–1.79	0.83
AST	1.97	1.32–2.92	< 0.001	1.05	0.64–1.71	0.849
Cirrhosis	0.82	0.55–1.21	0.313			
Diameter	1.13	1.07–1.19	< 0.001	1.03	0.96–1.10	0.375
GGT	2.19	1.49–3.23	< 0.001	1.39	0.87–2.21	0.174
Viral hepatitis						
No	ref					
HBV	0.76	0.49–1.18	0.225			
HCV	1.45	0.44–4.78	0.541			
HBV + HCV co-infection	1.06	0.14–7.87	0.951			
Number	1.24	0.81–1.90	0.317			
Nomogram-predicted MTM state	3.22	2.16–4.81	< 0.001	1.72	1.03–2.88	0.039
Sex	1.56	0.92–2.63	0.096			
Type of surgery						
Right hepatectomy	ref					
Left hepatectomy	0.77	0.45–1.29	0.316			
Extended hepatectomy	1.42	0.80–2.52	0.230			
Right or left lobectomy	0.96	0.55–1.67	0.887			
Right or left segmentectomy	0.47	0.20–1.07	0.073			
Method of surgery						
Open surgery	ref					
Laparoscopic surgery	1.45	0.97–2.16	0.069			

Nomogram-predicted MTM state including predicted MTM + HCC and predicted MTM- HCC. *AFP* Alpha-fetoprotein, *ALT* Alanine aminotransferase, *AST* Aspartate aminotransferase, *CI* confidence interval, *GGT* Gamma-glutamyl transferase, *HR* Hazard ratio, *MTM* + *HCC* Macrotrabecular-massive hepatocellular carcinoma

## Discussion

MTM + HCC, characterized by its highly aggressive biological behavior and poor prognosis [24], poses significant challenges in clinical strategies. In this study, we developed the DL habitat radiomics fusion nomogram based on Gd-EOB-DTPA-enhanced MRI, which demonstrated superior diagnostic performance compared to the CR model. Notably, patients stratified as MTM + HCC by the nomogram exhibited significantly shorter RFS than those predicted as MTM- HCC.

Our study confirmed that AFP is an independent predictor for identifying MTM + HCC, which is consistent with previous findings [25], indicating the high aggressiveness of MTM + HCC. Cheng et al [26] found that the presence of fat in the tumor was an independent predictor for identifying MTM + HCC, which was consistent with our study. Previous studies have demonstrated that fat in mass is more frequently observed in less aggressive HCC and is generally associated with a better prognosis [27–29]. As a highly aggressive subtype, MTM + HCC typically lacks this characteristic. Our study also revealed that elevated ALT levels serve as an independent predictor for identifying MTM + HCC. Previous studies demonstrated that elevated ALT levels are associated with adverse outcomes in HCC patients [30–32]. Elevated ALT levels, indicative of severe hepatic dysfunction, were associated with the aggressive nature of MTM + HCC.

Li et al [20] established a DL radiomics model based on dual-energy CT, achieving excellent diagnostic performance with an AUC of 0.89 in the external test set. Our study built various models based on Gd-EOB-DTPA-enhanced MRI, which is widely used in HCC diagnosis. In our study, the performance of the DL habitat radiomics fusion nomogram was superior to that of the CR model. Li et al [20] also found that the DL radiomics model showed better performance than the CR model. The CR model, integrating clinical and qualitative imaging features, was inherently constrained by subjective interpretation and limited variables.

In recent years, radiomics methods have shown high efficacy in identifying MTM + HCC. Zhang et al [33] constructed a clinical-radiomics model for MTM + HCC with an AUC of 0.805, but their single-center design limited generalizability. Zhang et al [34] developed a multiphasic CT model with an AUC of 0.836, while our DL-based approach better captures nonlinear patterns. He et al [35] developed a DL and radiomics model based on contrast-enhanced CT, achieving an AUC of 0.773 in the external test set. In contrast, our study utilized habitat radiomics, which more effectively captured the intratumoral spatial heterogeneity, achieving a higher AUC (0.858, 95% CI: 0.797–0.906) in the external test set.

There are some limitations to this study. Firstly, as a retrospective cohort, the study was inherently vulnerable to selection bias. Secondly, given the substantial acquisition heterogeneity across the three centers, no dedicated harmonization method was applied, which may limit the generalizability of our findings. Thirdly, the computational demands of our multi‑level fusion nomogram may hinder real‑time clinical use, and an end‑to‑end DL model was not employed to preserve interpretability. Finally, insufficient survival data precluded assessment of overall survival. Future research should aim to expand the sample size to comprehensively evaluate the predictive value of the nomogram in patients’ survival.

In conclusion, our study constructed a DL habitat radiomics fusion nomogram based on Gd-EOB-DTPA-enhanced MRI. The nomogram could serve as a robust non-invasive tool for preoperative MTM + HCC diagnosis. Future work should focus on validating our model in larger, more diverse cohorts and exploring its integration into clinical workflows to improve patient outcomes.

## Supplementary information

**Additional file 1:** **Table S1:** The 3.0-T MRI scan sequences and parameters for three medical systems. **Table S2:** Automatically segmentation performance of nnU-Net compared with ground truth in 254 HCC cases. **Table S3:** Multivariable analysis for identifying MTM+ HCC in the Clinical-radiologic model. **Table S4:** Performance of habitat radiomics models for predicting MTM+ HCC. **Table S5:** Performance of DL models for predicting MTM+ HCC. **Table S6:** Comparison of habitat radiomics models and DL models across different phases. **Table S7:** Comparison of habitat radiomics score and DL score between MTM+ and MTM- groups in the training, internal validation, and external test sets. **Table S8:** Multivariable analysis for identifying MTM+ HCC in the DL habitat radiomics fusion nomogram. **Table S9:** Type and method of surgery across the training, internal validation, and external test sets. **Fig. S1:** The loss curves and Dice scores of the nnU‑Net model in the arterial phase (**a**), portal venous phase (**b**), and hepatobiliary phase (**c**). **Fig. S2:** The Calinski-Harabasz index for different numbers of clusters. **Fig. S3:** The loss (**a**) and accuracy (**b**) curves in the training and validation sets of the 3D Swin Transformer model for arterial phase. **Fig. S4:** ROC curves of AP, PP, HBP and CP habitat radiomics models in the training (**a**), internal validation (**b**), and external test (**c**) sets. The ROC curves of the AP, PP, HBP and CP deep learning models in the training (**d**), internal validation (**e**), and external test (**f**) sets. ROC: Receiver operating characteristic; AUC: Area under the receiver operating characteristic curve; AP: Arterial phase; PP: Portal venous phase; HBP: Hepatobiliary phase; CP: Combined phase. **Fig. S5:**—The heatmaps of the selected features in the combined phase (CP) habitat radiomics model (**a**) and the CP deep learning model (**b**). The network plots of the selected features in the CP habitat radiomics model (**c**) and the CP deep learning model (**d**). AP: Arterial phase; PP: Portal venous phase; HBP: Hepatobiliary phase. **Fig. S6:** The heat map of the selected features in the DL habitat radiomics fusion nomogram. AFP: Alpha-fetoprotein; ALT: Alanine aminotransferase; DL: Deep learning. **Fig. S7:** The confusion matrix for the deep learning habitat fusion radiomics nomogram in the training (**a**), internal validation (**b**), and external test (**c**) sets. **Fig. S8:** The DL habitat fusion radiomics model interpretability using Shapley Additive Explanation methodology. Summary plots of features values against SHAP values and heatmap plots in the training (**a**), internal validation (**b**), and external test (**c**) sets. DL: Deep learning; ALT: Alanine aminotransferase; AFP: Alpha-fetoprotein.

## Data Availability

The data used or analyzed during the current study are available from the corresponding author upon reasonable request.

## Abbreviations

AFP: Alpha-fetoprotein
ALT: Alanine transferase
AP: Arterial phase
APHE: Arterial phase hyperenhancement
AST: Aspartate transaminase
AUC: Area under the (receiver operating characteristic) curve
CI: Confidence interval
CP: Combined-phase
CR: Clinical-radiologic
CT: Computed tomography
DL: Deep learning
Gd-EOB-DTPA: Gadolinium-ethoxybenzyl-diethylenetriamine pentaacetic acid
GGT: γ-glutamyl transferase
HBP: Hepatobiliary phase
HCC: Hepatocellular carcinoma
HR: Hazard ratio
MRI: Magnetic resonance imaging
MTM+: Macrotrabecular-massive
PP: Portal venous phase
RFS: Recurrence-free survival
SHAP: Shapley additive explanations
