# Supplementary material for: Interpretable prediction of macrotrabecular-massive HCC and recurrence-free survival by integrating MRI LI-RADS features with deep learning habitat radiomics
Source: Eur Radiol Exp. 2026 Sep 8;10:121. doi: 10.1186/s41747-026-00795-y (PMC13554023; doi:10.1186/s41747-026-00795-y)
Supplement: Supplementary file 1 [file 41747_2026_795_MOESM1_ESM.pdf]

# Interpretable prediction of macrotrabecular-massive HCC and recurrence-free survival by integrating MRI LI-RADS features with deep learning habitat radiomics

## ELECTRONIC SUPPLEMENTARY MATERIAL

*Eur Radiol Exp (2026) Zhang M, Wu Q, Zhu M et al.*

**Appendix**

**Appendix S1: Definition of qualitative features**

Infiltrative appearance was defined as observation with non-circumscribed margin thought to represent malignancy with permeative growth pattern. Fat in mass was defined as decreased out-of-phase T1WI compared with in-phase T1WI. Blood product in mass was defined as hyperintensity in T1WI and out-of phase. Enhancement pattern was recorded as nonrim APHE and rim APHE. Nonrim APHE was defined as nonrim-like enhancement in the arterial phase, which is clearly greater in its entirety or in part compared to the liver. Rim APHE was defined as arterial phase enhancement which was most pronounced in the observation periphery. Washout was defined as the reduction in enhancement compared to liver tissue from earlier to later phase. Non-peripheral washout mainly refers to the washout that occurs in non-peripheral tumor areas. The counterpart of nonperipheral washout is peripheral washout which is most pronounced in observation periphery. Capsule, defined as a peripheral rim-like border was divided into two types: (1) enhancing capsule which presented hyperenhancement in the portal venous or delayed phase; (2) non-enhancing capsule, which showed no enhancing rim-like in all phases. Arterial phase peritumoral enhancement was defined as an irregular enhancement outside the tumor margin in the arterial phase, becoming isointense in the delayed phase. Intratumoural artery was defined as an artery within the tumor observed in the arterial phase. Substantial necrosis was defined as a central area of high signal intensity on fat-suppressed turbo-spin-echo T2WI without enhancement on postcontrast T1WI. We have classified the degree of necrosis into two levels(＞20% and ＞50%) based on the proportion of the necrotic area in the tumor volume. Satellite nodule was defined as a lesion smaller than 2 cm that appeared within 2 cm around the tumor. Central scar, primarily composed of fibrous tissue, was defined as a central T2WI hyperintensity or T1 hypointensity, accompanied by a hyperintensity in the hepatobiliary phase.

Peritumoral hypointensity in the Hepatobiliary phase(HBP) was defined as hypointensity area of hepatic parenchyma around the tumor in HBP. Gadolinium-enhanced clouding was defined as a cloud-like appearance in the central region of the tumor in hepatobiliary phase. LI-RADS category referred to the Liver Imaging Reporting and Data System, version 2018.

**Appendix S2: Automatic segmentation using nnU-Net**

The nnU-Net framework (https://github.com/MIC-DKFZ/nnUNet) was employed for automatic segmentation. 353 cases were randomly split into training (n=282) and validation sets(n=71). The model was trained using the default 3D full-resolution configuration of nnU-Net. Data augmentation techniques, including random rotations, scaling, and intensity normalization, were applied during training to improve model robustness. The network was optimized using stochastic gradient descent (SGD) with Nesterov momentum (momentum = 0.99) and an initial learning rate of 0.01, following the default nnU-Net configuration. Training was performed for 1000 epochs, and the weights of the final checkpoint were saved for subsequent automatic segmentation prediction.

The nnU-Net model achieved stable convergence during training. It obtained the best exponential moving average (EMA) pseudo dice scores of 0.841, 0.875, and 0.890 during training, and mean validation dice coefficients were 0.796 ,0.805, and 0.822 in the AP, PP and HBP, respectively. The loss curves and Dice scores indicated effective learning and good generalization (Figure S2). The segmentation results demonstrated that nnU-Net could accurately delineate the target structures, providing reliable performance for HCC automatic segmentation.

**Appendix S3：DL and habitat radiomics feature selection**

The detailed procedure was outlined below. The Synthetic Minority Oversampling Technique

(SMOTE) was used to balance positive and negative samples in the training data set. The Z-score normalization was applied on the feature matrix. After normalization, the relevance of each feature pair was compared. If the Pearson Correlation Coefficient (PCC) of the feature pair was larger than 0.80, one of them was removed. After this process, the dimension of the feature space was reduced, and all features were independent of each other. Analysis of variance (ANOVA) was employed for feature selection by ranking features based on their F-values, which indicate the strength of their association with the target variable.

**Appendix S4：Comparison of DL and habitat radiomics models**

DeLong test comparisons revealed that, after applying the Bonferroni correction (significance threshold set at $P$<0.0083), the CP DL model significantly outperformed the AP DL model in the internal validation set (0.712 [95% CI: 0.618-0.805] vs. 0.543 [95% CI: 0.433-0.654], $P$ = 0.004). Additionally, the CP DL model showed significantly higher AUCs than the PP DL model in the training set(0.740 [95% CI: 0.676-0.804] vs. 0.635 [95% CI: 0.566-0.704], $P$ = 0.005).

**Table S1:The 3.0T MRI scan sequences and parameters for three medical systems**

| Sequence | Category | TR(ms) | TE(ms) | FOV (mm) | Thickness (mm) | Fat saturation | Breath-hold |
|---|---|---|---|---|---|---|---|
| Siemens Medical Systems | | | | | | | |
| T1WI in/ opposed phase | 3D VIBE | 4.11 | 2.47/1.24 | 400*400 | 3.5 | No | Yes |
| T1WI-fs | 3D VIBE | 4.11 | 1.24 | 400*400 | 3.5 | Yes | Yes |
| T2WI-fs | TSE | 3000 | 82 | 400*400 | 6.5 | Yes | Yes |
| DWI | Ep2d | 5600 | 58 | 400*400 | 6.5 | Yes | No |
| T1WI-fs (AP) | 3D VIBE | 4.15 | 2.01 | 400*400 | 3.5 | Yes | Yes |
| T1WI-fs (PP) | 3D VIBE | 4.15 | 2.01 | 400*400 | 3.5 | Yes | Yes |
| T1WI-fs (HBP) | 3D VIBE | 4.15 | 2.01 | 400*400 | 3.5 | Yes | Yes |
| GE Medical Systems | | | | | | | |
| T1WI in/ opposed phase | Fast SPGR | 4.6 | 1.2 | 400*360 | 2.5 | No | Yes |
| T1WI-fs | LAVA-Flex | 4.5 | 1.4 | 380*342 | 2.5 | Yes | Yes |
| T2WI-fs | FRFSE-XL | 7500 | 86.2 | 400*300 | 7 | Yes | Yes |
| DWI | DW EPI | 10000 | 57.1 | 400*320 | 2 | Yes | No |
| T1WI-fs (AP) | LAVA-Flex | 4.6 | 1.7 | 380*342 | 2.5 | Yes | Yes |
| T1WI-fs (PP) | LAVA-Flex | 4.6 | 1.7 | 380*342 | 2.5 | Yes | Yes |
| T1WI-fs (HBP) | LAVA-Flex | 4.6 | 1.7 | 380*342 | 2.5 | Yes | Yes |
| Philips Medical Systems | | | | | | | |
| T1WI in/ opposed phase | mDIXON | 3.7 | 1.32 | 400*300 | 6 | No | Yes |
| T1WI-fs | mDIXON | 3.7 | 1.32 | 400*300 | 6 | Yes | Yes |
| T2WI-fs | TSE | 448 | 70 | 320*364 | 4 | Yes | Yes |

| | | | | | | | |
|---|---|---|---|---|---|---|---|
| DWI | DW EPI | 1783 | 55 | 300*360 | 5 | Yes | No |
| T1WI-fs (AP) | mDIXON | 3.8 | 1.4 | 400*300 | 3 | Yes | Yes |
| T1WI-fs (PP) | mDIXON | 3.8 | 1.4 | 400*300 | 3 | Yes | Yes |
| T1WI-fs (HBP) | mDIXON | 3.8 | 1.4 | 400*300 | 3 | Yes | Yes |

FOV: field of view; 3D VIBE: a three-dimensional volume interpolated breath-hold examination; Ep2d: a two-dimensional echo-planar technique; Fast SPGR: Fast Spoiled Gradient Recalled Echo; LAVA-Flex: Liver Acquisition with Volume Acceleration-Flexible; FRFSE-XL: Fast Recovery Fast Spin Echo-eXtended Echo Train Length; DW EPI: Diffusion-Weighted Echo Planar Imaging; mDIXON; modified Dixon; TSE; turbo spin-echo; fs: fat suppression; AP:arterial phase; PP:portal venous phase; HBP: hepatobiliary phase.

**Table S2:Automatically segmentation performance of nnU-Net compared with ground truth in 254 HCC cases**

| Phase | DSC | | | HD95(mm) | | |
|---|---|---|---|---|---|---|
| | ＞5cm | 3-5cm | ≤3cm | ＞5cm | 3-5cm | ≤3cm |
| AP | 0.921 | 0.915 | 0.803 | 8.809 | 12.971 | 24.815 |
| PP | 0.910 | 0.874 | 0.846 | 15.080 | 9.964 | 16.020 |
| HBP | 0.935 | 0.979 | 0.844 | 11.002 | 4.573 | 19.163 |

AP: arterial phase; PP: portal venous phase; HBP: hepatobiliary phase; HD95: 95th percentile Hausdorff distance (mm); DSC: the Dice similarity coefficient.

**Table S3: Multivariable analysis for identifying MTM+ HCC in the Clinical-radiologic model**

| Variables | Coefficients | OR[95%CI] | *p*-value |
|---|---|---|---|
| ALT | 0.890 | 2.435[1.242-4.772] | 0.010 |
| AFP | 1.043 | 2.839[1.584-5.085] | ＜0.001 |
| Maximum tumor diameter | 0.578 | 1.782[1.329-2.389] | ＜0.001 |
| Fat in mass | -1.841 | 0.159[0.065-0.389] | ＜0.001 |
| Capsule | | | 0.098 |
| Enhancing capsule | 0.578 | 1.782[0.944-3.366] | 0.075 |
| Non-enhancing capsule | 0.851 | 2.343[0.953-5.759] | 0.064 |
| Peritumoural hypointensity in the HBP | 0.984 | 2.675[1.234-5.799] | 0.013 |

MTM+ HCC:Macrotrabecular-massive hepatocellular carcinoma; OR = odds ratio; CI: confidence interval;
ALT:alanine aminotransferase; AFP:alpha-fetoprotein.

**Table S4: Performance of habitat radiomics models for predicting MTM+ HCC**

| Variables | | AP | PP | HBP | CP |
|---|---|---|---|---|---|
| Training set | AUC | 0.759 | 0.774 | 0.775 | 0.802 |
| | [95%CI] | [0.695-0.823] | [0.716-0.833] | [0.718-0.832] | [0.747-0.858] |
| | Brier score | 0.192 | 0.203 | 0.201 | 0.179 |
| | ECE | 0.182 | 0.188 | 0.178 | 0.155 |
| | Calibration slope | 1.474 | 1.847 | 1.354 | 1.233 |
| | F1 score | 0.584[0.503-0.658] | 0.585[0.510-0.649] | 0.625[0.542-0.702] | 0.624[0.548-0.694] |
| | accuracy | 0.724 | 0.645 | 0.763 | 0.730 |
| | sensitivity | 0.670[0.571-0.758] | 0.864[0.785-0.929] | 0.682[0.580-0.783] | 0.773[0.686-0.854] |
| | specificity | 0.745[0.685-0.799] | 0.556[0.486-0.618] | 0.796[0.744-0.851 | 0.713[0.654-0.777] |
| Internal validation set | AUC | 0.631 | 0.565 | 0.669 | 0.687 |
| | [95%CI] | [0.525-0.737] | [0.449-0.680] | [0.566-0.772] | [0.585-0.789] |
| | Brier score | 0.235 | 0.233 | 0.223 | 0.218 |
| | ECE | 0.211 | 0.200 | 0.203 | 0.190 |
| | Calibration slope | 0.525 | 0.547 | 0.840 | 0.402 |
| | F1 score | 0.519[0.396-0.634] | 0.413[0.278-0.533] | 0.495[0.369-0.606] | 0.519[0.395-0.632] |
| | accuracy | 0.618 | 0.588 | 0.595 | 0.618 |
| | sensitivity | 0.794[0.640-0.923] | 0.559[0.387-0.724] | 0.765[0.606-0.903] | 0.794[0.643-0.921] |
| | specificity | 0.557[0.466-0.657] | 0.598[0.500-0.692] | 0.536[0.444-0.636] | 0.557[0.456-0.651] |
| External test set | AUC | 0.606 | 0.728 | 0.661 | 0.681 |
| | [95%CI] | [0.476-0.736] | [0.605-0.852] | [0.541-0.781] | [0.553-0.808] |
| | Brier score | 0.355 | 0.180 | 0.183 | 0.145 |
| | ECE | 0.460 | 0.242 | 0.240 | 0.140 |
| | Calibration slope | 0.676 | 2.191 | 1.539 | 0.913 |
| | F1 score | 0.385[0.192-0.531] | 0.506[0.364-0.624] | 0.473[0.293-0.621] | 0.484[0.321-0.635] |
| | accuracy | 0.814 | 0.750 | 0.831 | 0.814 |
| | sensitivity | 0.345[0.172-0.517] | 0.759[0.565-0.897] | 0.448[0.264-0.643] | 0.517[0.325-0.705] |
| | specificity | 0.909[0.861-0.953] | 0.748[0.669-0.817] | 0.909[0.850-0.951] | 0.874[0.808-0.923] |

MTM+ HCC: Macrotrabecular-massive hepatocellular carcinoma; AUC = area under the receiver operating characteristic curve; CI: confidence interval; ECE: expected calibration error; AP: arterial phase; PP: portal venous phase; HBP: hepatobiliary phase; CP: combined phase.

**Table S5: Performance of DL models for predicting MTM+ HCC**

| Variables | | AP | PP | HBP | CP |
|---|---|---|---|---|---|
| Training set | AUC | 0.653 | 0.635 | 0.661 | 0.740 |
| | [95%CI] | [0.590-0.716] | [0.566-0.703] | [0.590-0.733] | [0.676-0.804] |
| | Brier score | 0.240 | 0.242 | 0.235 | 0.200 |
| | ECE | 0.214 | 0.224 | 0.210 | 0.176 |
| | Calibration slope | 0.860 | 0.764 | 0.916 | 1.045 |
| | F1 score | 0.517[0.444-0.582] | 0.421[0.318-0.510] | 0.521[0.433-0.613] | 0.559[0.478-0.636] |
| | accuracy | 0.520 | 0.711 | 0.740 | 0.694 |
| | sensitivity | 0.886[0.800-0.944] | 0.364[0.264-0.473] | 0.488[0.380-0.598] | 0.670[0.562-0.767] |
| | specificity | 0.370[0.306-0.438] | 0.852[0.797-0.896] | 0.843[0.787-0.888] | 0.704[0.638-0.764] |
| Internal validation set | AUC | 0.543 | 0.620 | 0.665 | 0.712 |
| | [95%CI] | [0.433-0.654] | [0.503-0.736] | [0.556-0.774] | [0.618-0.805] |
| | Brier score | 0.259 | 0.250 | 0.238 | 0.207 |
| | ECE | 0.244 | 0.275 | 0.244 | 0.191 |
| | Calibration slope | 0.334 | 0.755 | 0.823 | 0.908 |
| | F1 score | 0.443[0.336-0.547] | 0.429[0.264-0.560] | 0.475[0.323-0.604] | 0.515[0.403-0.616] |
| | accuracy | 0.366 | 0.695 | 0.679 | 0.512 |
| | sensitivity | 0.971[0.847-0.999] | 0.441[0.272-0.621] | 0.559[0.379-0.728] | 1.000[0.897-1.000] |
| | specificity | 0.155[0.089-0.242] | 0.784[0.688-0.861] | 0.722[0.621-0.808] | 0.340[0.247-0.443] |
| External test set | AUC | 0.580 | 0.582 | 0.593 | 0.725 |
| | [95%CI] | [0.465-0.696] | [0.461-0.703] | [0.455-0.732] | [0.624-0.825] |
| | Brier score | 0.257 | 0.233 | 0.204 | 0.182 |
| | ECE | 0.339 | 0.310 | 0.272 | 0.234 |
| | Calibration slope | 0.580 | 0.834 | 1.031 | 1.059 |
| | F1 score | 0.316[0.121-0.500] | 0.341[0.197-0.468] | 0.393[0.226-0.556] | 0.404[0.272-0.518] |
| | accuracy | 0.849 | 0.686 | 0.802 | 0.640 |
| | sensitivity | 0.207[0.080-0.397] | 0.483[0.294-0.675] | 0.379[0.207-0.577] | 0.724[0.528-0.873] |
| | specificity | 0.979[0.940-0.996] | 0.727[0.646-0.798] | 0.881[0.825-0.935] | 0.622[0.538-0.702] |

DL: Deep learning; MTM+ HCC: Macrotrabecular-massive hepatocellular carcinoma; AUC = area under the receiver operating characteristic curve; CI: confidence interval; ECE: expected calibration error; AP: arterial phase; PP: portal venous phase; HBP: hepatobiliary phase; CP: combined phase.

## Table S6:Comparison of habitat radiomics models and DL models across different phases

| Variables | Training set | | | Internal validation set | | | External test set | | |
|---|---|---|---|---|---|---|---|---|---|
| | AUC | *z*-value | *p*-value | AUC | *z*-value | *p*-value | AUC | *z*-value | *p*-value |
| Habitat radiomics model | | | | | | | | | |
| AP vs PP | 0.759 vs 0.774 | 0.498 | 0.619 | 0.631 vs 0.565 | 0.972 | 0.331 | 0.606 vs 0.728 | 1.384 | 0.167 |
| AP vs HBP | 0.759 vs 0.775 | 0.473 | 0.636 | 0.631 vs 0.669 | 0.661 | 0.509 | 0.606 vs 0.661 | 0.745 | 0.456 |
| AP vs CP | 0.759 vs 0.802 | 1.685 | 0.092 | 0.631 vs 0.687 | 1.035 | 0.301 | 0.606 vs 0.680 | 1.026 | 0.305 |
| PP vs HBP | 0.774 vs 0.775 | 0.0167 | 0.987 | 0.565 vs 0.669 | 2.03 | 0.042 | 0.728 vs 0.661 | 0.848 | 0.396 |
| PP vs CP | 0.774 vs 0.802 | 1.176 | 0.240 | 0.565 vs 0.687 | 2.575 | 0.010 | 0.661 vs 0.680 | 0.794 | 0.427 |
| HBP vs CP | 0.775 vs 0.802 | 0.987 | 0.324 | 0.669 vs 0.687 | 0.386 | 0.700 | 0.661 vs 0.680 | 0.276 | 0.783 |
| DL model | | | | | | | | | |
| AP vs PP | 0.653 vs 0.635 | 0.392 | 0.695 | 0.543 vs0.620 | 0.926 | 0.354 | 0.580 vs 0.582 | 0.0166 | 0.987 |
| AP vs HBP | 0.653 vs 0.661 | 0.190 | 0.849 | 0.543 vs0.665 | 1.429 | 0.153 | 0.580 vs 0.593 | 0.127 | 0.899 |
| AP vs CP | 0.653 vs 0.740 | 2.213 | 0.027 | 0.543 vs0.712 | 2.900 | 0.004 | 0.580 vs0.725 | 2.21 | 0.027 |
| PP vs HBP | 0.635 vs 0.661 | 0.508 | 0.611 | 0.620 vs 0.665 | 0.514 | 0.608 | 0.582 vs 0.593 | 0.108 | 0.914 |
| PP vs CP | 0.635 vs 0.740 | 2.819 | 0.005 | 0.620 vs 0.712 | 1.299 | 0.194 | 0.582 vs 0.725 | 2.152 | 0.031 |
| HBP vs CP | 0.661 vs 0.740 | 1.944 | 0.052 | 0.665 vs0.712 | 0.668 | 0.504 | 0.593 vs 0.725 | 1.528 | 0.126 |

DL: Deep learning; AUC = area under the receiver operating characteristic curve;
AP: arterial phase; PP: portal venous phase; HBP: hepatobiliary phase; CP: combined phase;
Bonferroni correction was applied with a significance threshold of $P < 0.0083$.

**Table S7: Comparison of habitat radiomics score and DL score between MTM+ and MTM- groups in the training, internal validation, and external test sets**

| Variables | Training set | | | Internal validation set | | | External test set | | |
|---|---|---|---|---|---|---|---|---|---|
| | MTM+ HCC | MTM- HCC | *p*-value | MTM+ HCC | MTM- HCC | *p*-value | MTM+ HCC | MTM- HCC | *p*-value |
| Habitat radiomics score | 0.591 [0.433,0.777] | 0.334 [0.277,0.463] | ＜0.001 | 0.492 [0.391,0.647] | 0.362 [0.293,0.515] | 0.001 | 0.373 [0.244,0.512] | 0.266 [0.217,0.334] | 0.002 |
| DL score | 0.569 [0.414,0.763] | 0.377 [0.282,0.523] | ＜0.001 | 0.478 [0.407,0.697] | 0.415 [0.281,0.506] | ＜0.001 | 0.518 [0.358,0.677] | 0.369 [0.245,0.476] | ＜0.001 |

DL: Deep learning; MTM+ HCC: Macrotrabecular-massive hepatocellular carcinoma.

**Table S8: Multivariable analysis for identifying MTM+ HCC in the DL habitat radiomics fusion nomogram**

| Variables | Coefficients | OR[95%CI] | *p*-value |
|---|---|---|---|
| ALT | 0.883 | 2.419[1.097-5.336] | 0.029 |
| AFP | 1.272 | 3.569[1.779-7.160] | ＜0.001 |
| Fat in mass | -1.907 | 0.149[0.053-0.414] | ＜0.001 |
| DL score | 1.118 | 3.060[2.092-4.474] | ＜0.001 |
| Habitat radiomics score | 1.270 | 3.561[2.420-5.241] | ＜0.001 |

MTM+ HCC:Macrotrabecular-massive hepatocellular carcinoma; OR = odds ratio; CI: confidence interval; ALT:alanine aminotransferase; AFP:alpha-fetoprotein; DL: deep learning.

**Table S9: Type and method of surgery across the training, internal validation, and external test sets**

| Characteristics | Training set($n$ = 262) Total | MTM+ HCC ($n$ = 83) | MTM- HCC ($n$ = 179) | $p$-value | Internal validation set($n$ = 109) | External test set($n$ = 165) | $p$-value[a] | $p$-value[b] |
|---|---|---|---|---|---|---|---|---|
| Type of surgery | | | | 0.546 | | | 0.131 | 0.796 |
| Right hepatectomy | 76(29.0) | 23(27.7) | 53(29.6) | | 31(28.4) | 41(24.8) | | |
| Left hepatectomy | 68(26.0) | 21(25.3) | 47(26.3) | | 27(24.8) | 36(21.8) | | |
| Extended hepatectomy | 37(14.1) | 14(16.9) | 23(12.8) | | 14(12.8) | 21(12.7) | | |
| Right or left lobectomy | 52(19.8) | 13(15.7) | 39(21.8) | | 21(19.3) | 34(20.6) | | |
| Right or left segmentectomy | 29(11.1) | 12(14.4) | 17(9.5) | | 16(14.7) | 33(20.0) | | |
| Method of surgery | | | | 0.152 | | | 0.253 | 0.338 |
| Open surgery | 170(64.9) | 59(71.1) | 111(62.0) | | 71(65.1) | 98(59.4) | | |
| Laparoscopic surgery | 92(35.1) | 24(28.9) | 68(38.0) | | 38(34.9) | 67(40.6) | | |

Note.Data are numbers of patients, with percentages in parentheses.
MTM+ HCC:Macrotrabecular-massive hepatocellular carcinoma;
$p$-value < 0.05 indicates a significant difference between MTM+ HCC and MTM- HCC in the training set.
$p$-value$^a$ < 0.05 indicates a significant difference between the training and external test set.
$p$-value$^b$ < 0.05 indicates a significant difference between the internal validation and external test set.

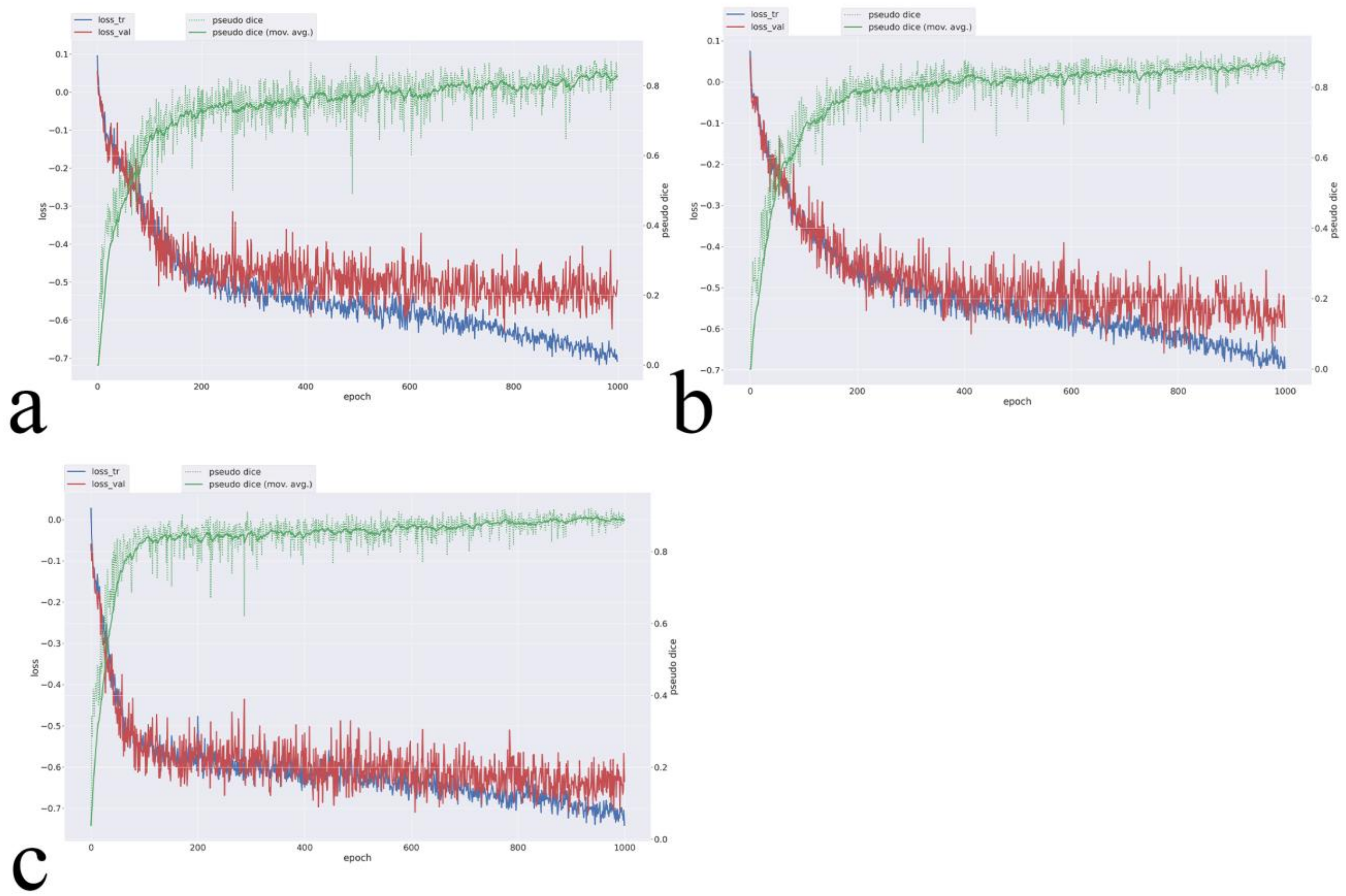


**Fig. S1:** The loss curves and Dice scores of the nnU-Net model in the arterial phase **(a)**, portal venous phase **(b)**, and hepatobiliary phase **(c)**.

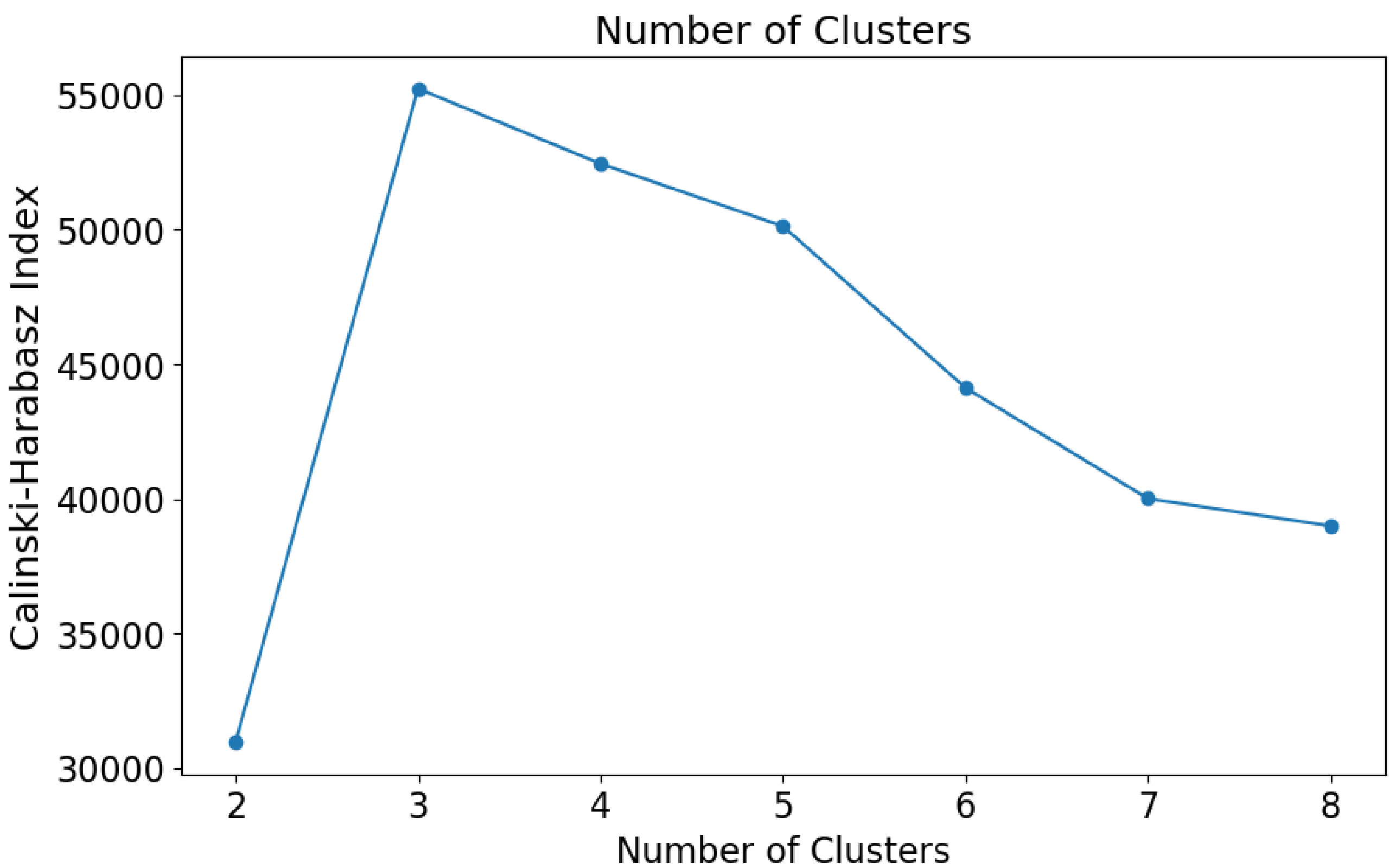


**Fig. S2:** The Calinski-Harabasz index for different numbers of clusters.

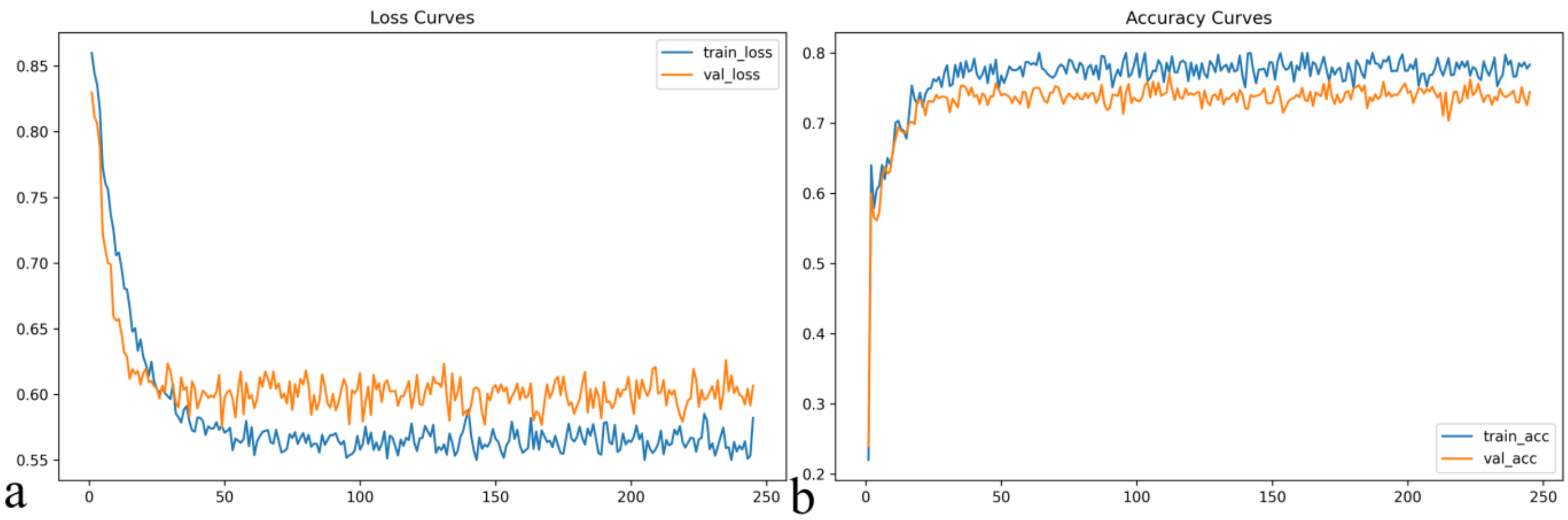


**Fig. S3:** The loss **(a)** and accuracy **(b)** curves in the training and validation sets of the 3D Swin Transformer model for arterial phase.

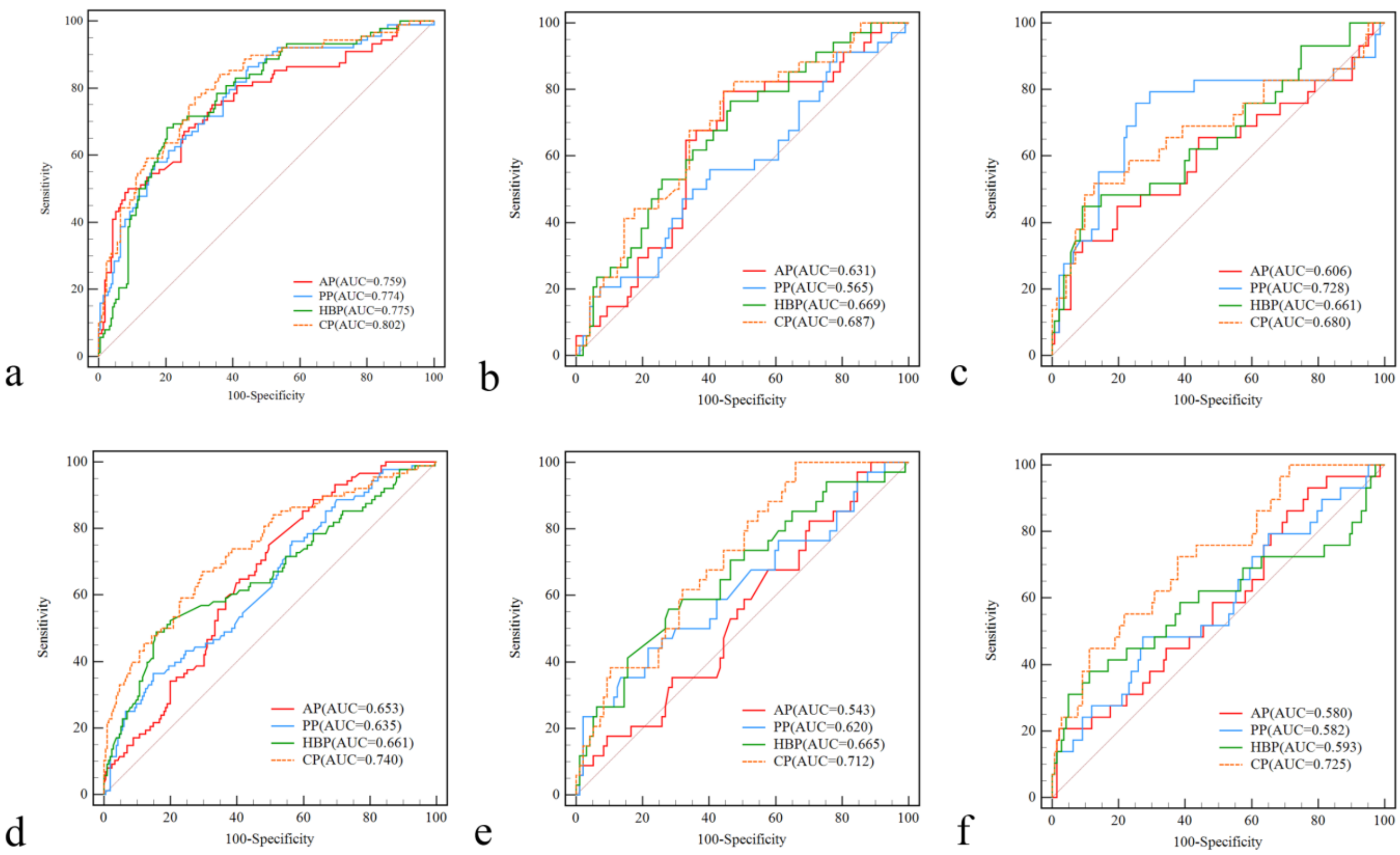


**Fig. S4:** ROC curves of AP, PP, HBP and CP habitat radiomics models in the training **(a)**, internal validation **(b)**, and external test **(c)** sets. The ROC curves of the AP, PP, HBP and CP deep learning models in the training **(d)**, internal validation **(e)**, and external test **(f)** sets. ROC: Receiver operating characteristic; AUC: Area under the receiver operating characteristic curve; AP: Arterial phase; PP: Portal venous phase; HBP: Hepatobiliary phase; CP: Combined phase.

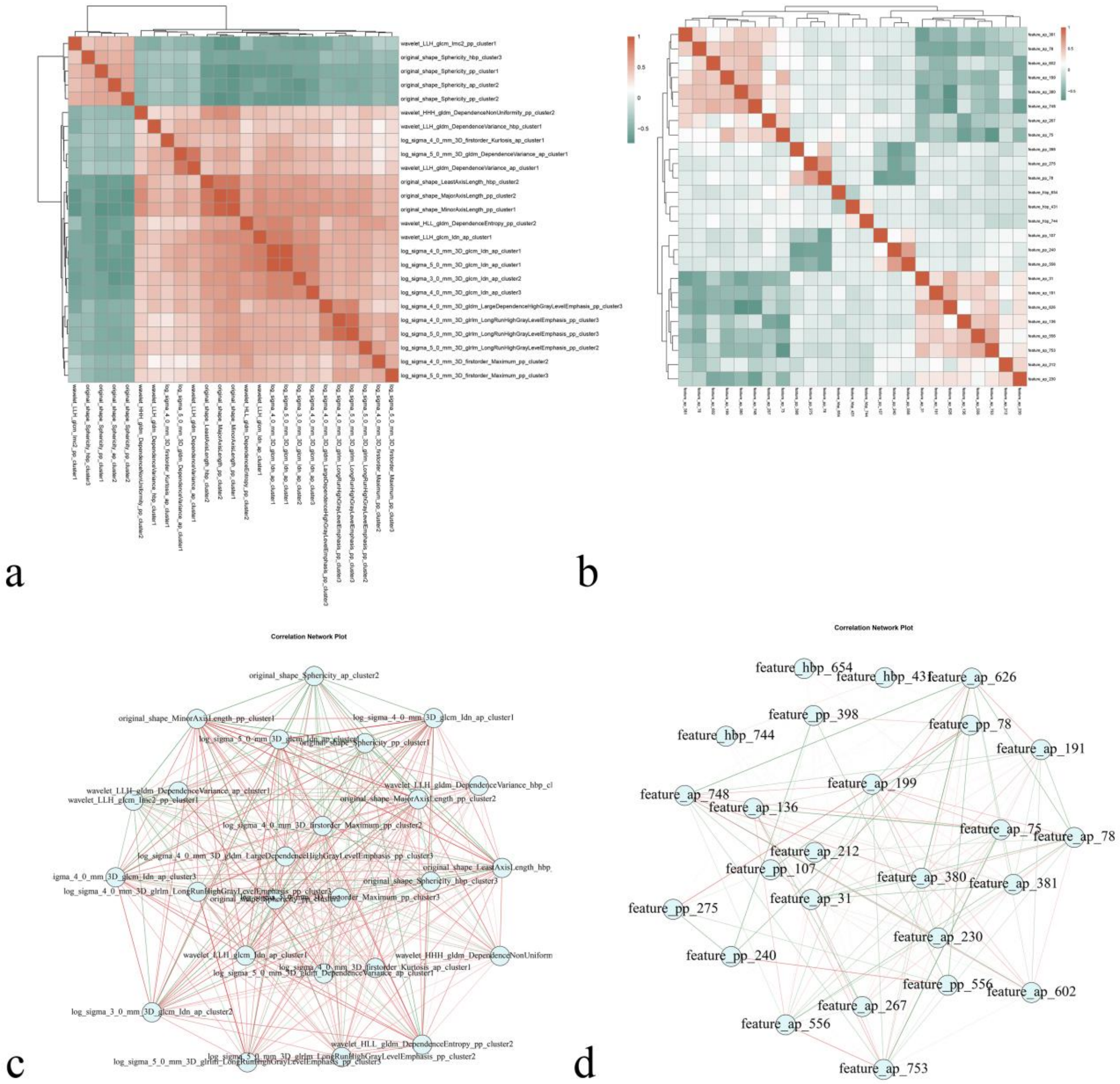


**Fig. S5**—The heatmaps of the selected features in the combined phase (CP) habitat radiomics model **(a)** and the CP deep learning model **(b)**. The network plots of the selected features in the CP habitat radiomics model **(c)** and the CP deep learning model **(d)**. AP: Arterial phase; PP: Portal venous phase; HBP: Hepatobiliary phase.

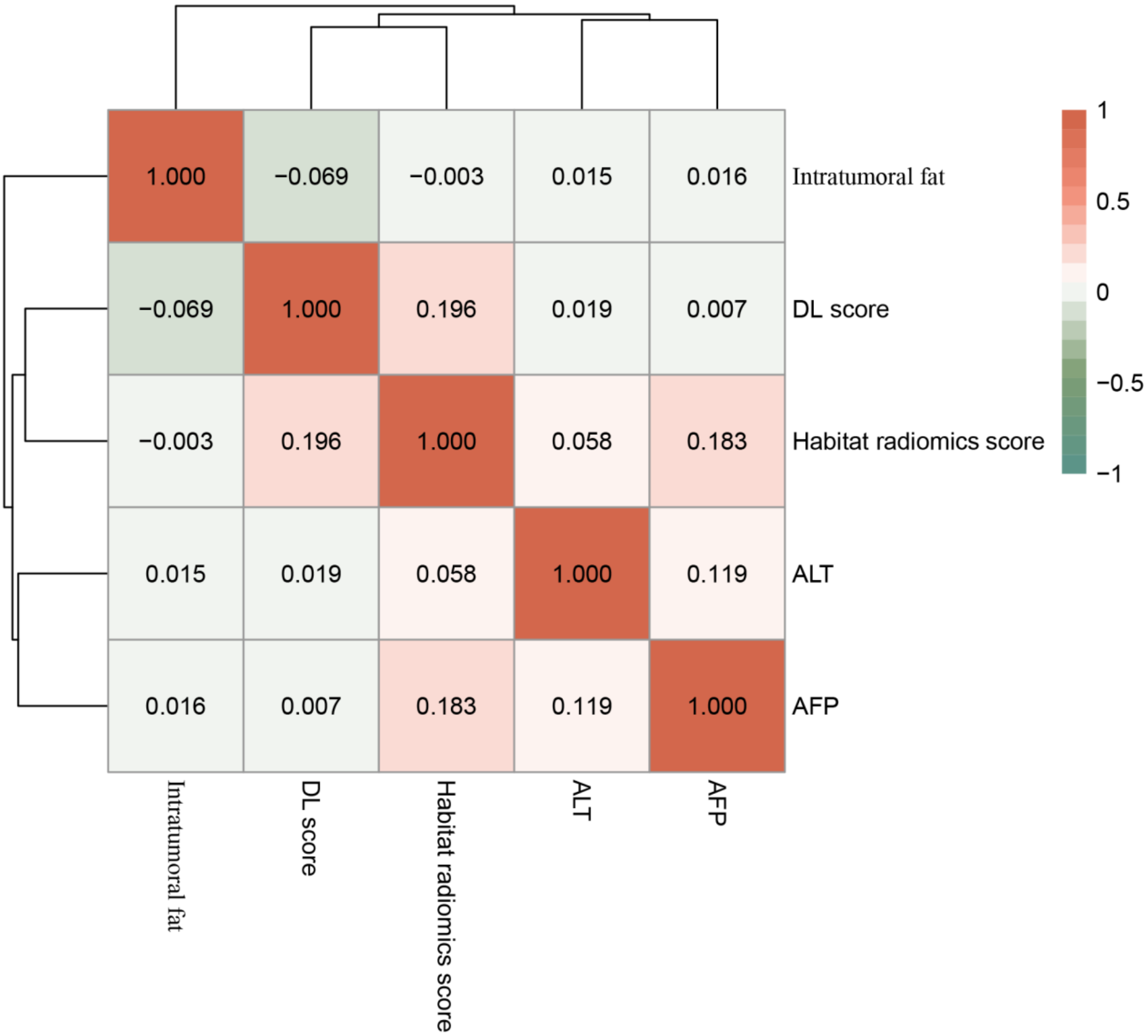


**Fig. S6:** The heat map of the selected features in the DL habitat radiomics fusion nomogram. AFP: Alpha-fetoprotein; ALT: Alanine aminotransferase; DL: Deep learning.

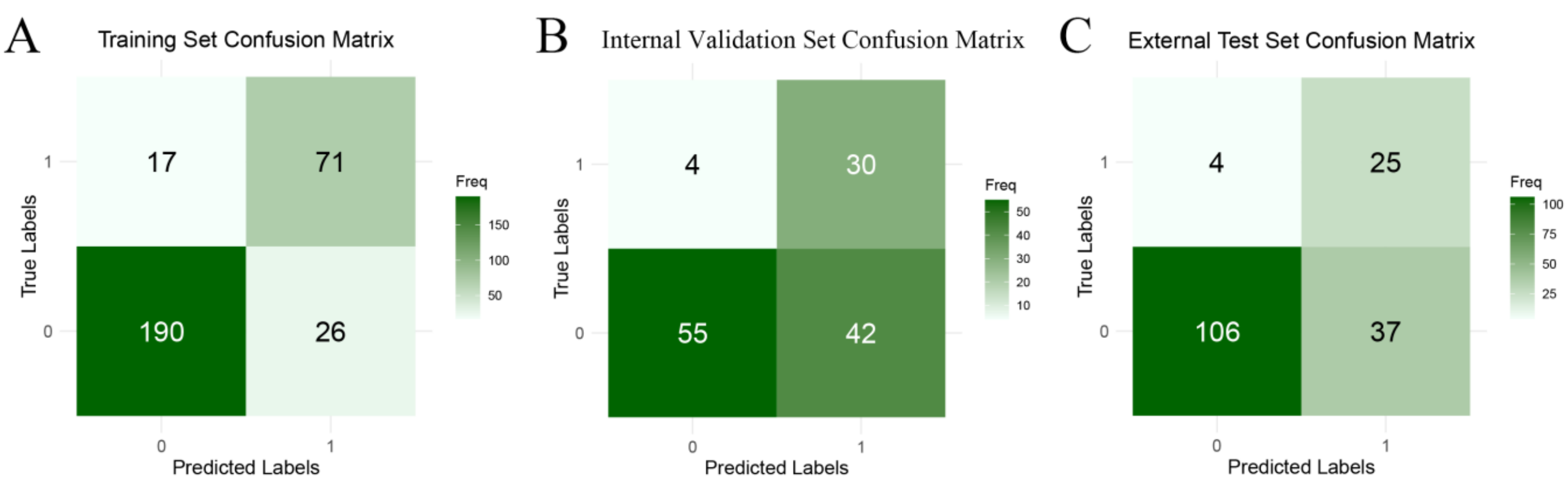


**Fig. S7:** The confusion matrix for the deep learning habitat fusion radiomics nomogram in the training **(a)**, internal validation **(b)**, and external test **(c)** sets.

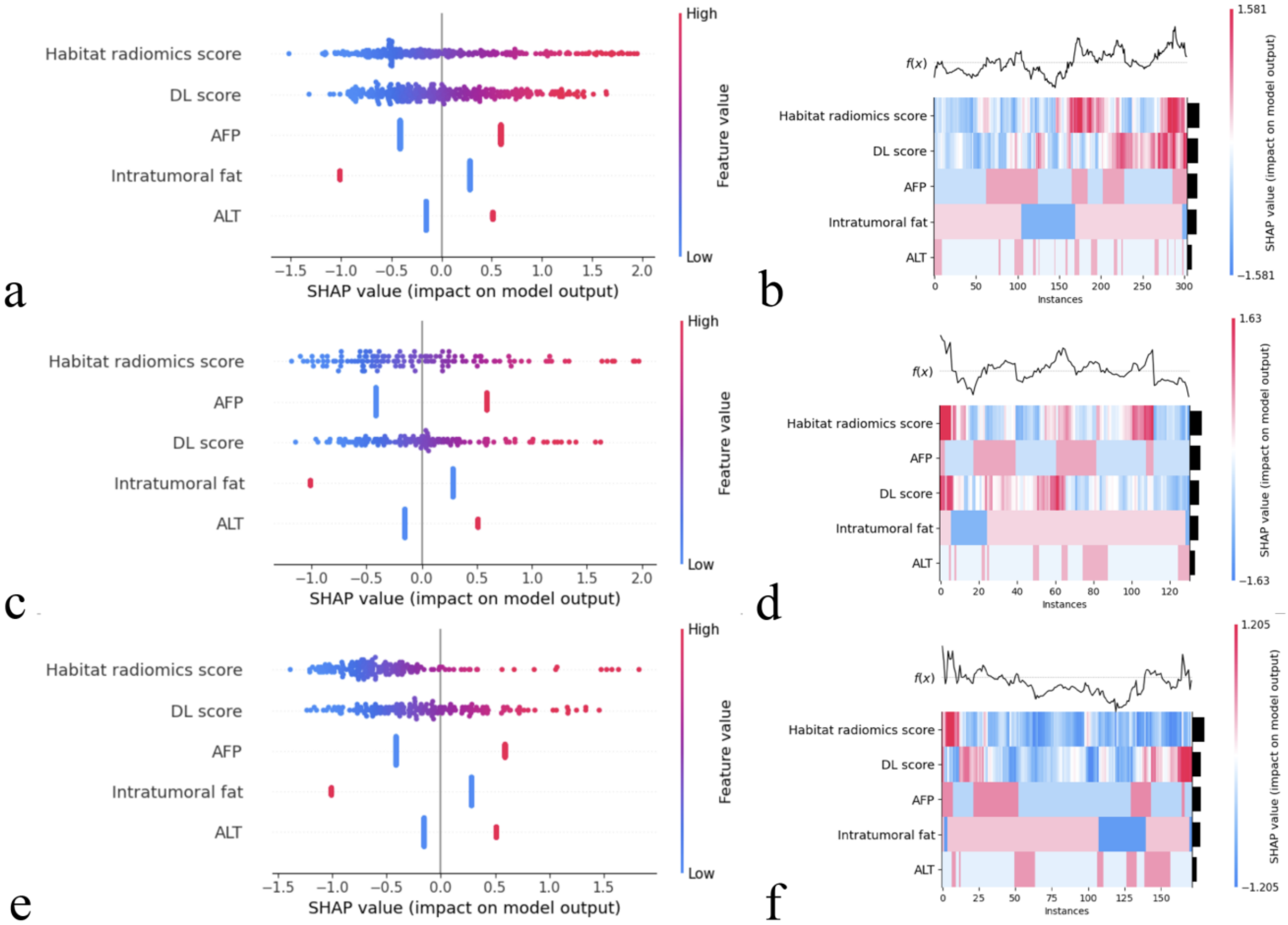


**Fig. S8**: The DL habitat fusion radiomics model interpretability using Shapley Additive Explanation methodology. Summary plots of features values against SHAP values and heatmap plots in the training **(a)**, internal validation **(b)**, and external test **(c)** sets. DL: Deep learning; ALT: Alanine aminotransferase; AFP: Alpha-fetoprotein.
